# GelMA/PEDOT:PSS Composite Conductive Hydrogel-Based Generation and Protection of Cochlear Hair Cells through Multiple Signaling Pathways

**DOI:** 10.3390/biom14010095

**Published:** 2024-01-11

**Authors:** Fei Tan, Xuran Li, Xiao Li, Maoxiang Xu, Khawar Ali Shahzad, Lei Hou

**Affiliations:** 1 Department of ORL-HNS, Shanghai Fourth People’s Hospital, School of Medicine, Tongji University, Shanghai 200070, China; 2111599@tongji.edu.cn (X.L.); xumaoxiang@tongji.edu.cn (M.X.); 23310050@tongji.edu.cn (K.A.S.); 2 Plasma Medicine and Surgical Implants Center, School of Medicine, Tongji University, Shanghai 200070, China; 3 Department of ORL-HNS, The Royal College of Surgeons in Ireland, D02 YN77 Dublin, Ireland; 4 Department of ORL-HNS, The Royal College of Surgeons of England, London WC2A 3PE, UK; 5 State Key Laboratory for Modification of Chemical Fibers and Polymer Materials, College of Chemistry, Chemical Engineering and Biotechnology & Center for Advanced Low-Dimension Materials, Donghua University, Shanghai 200051, China; 2220895@mail.dhu.edu.cn (X.L.); houlei@dhu.edu.cn (L.H.)

**Keywords:** conductive hydrogel, hair cell, hair cell regeneration, apoptosis, ferroptosis, cochlear implant, electroacoustic stimulation, GelMA, PEDOT:PSS

## Abstract

Recent advances in cochlear implantology are exemplified by novel functional strategies such as bimodal electroacoustic stimulation, in which the patient has intact low-frequency hearing and profound high-frequency hearing pre-operatively. Therefore, the synergistic restoration of dysfunctional cochlear hair cells and the protection of hair cells from ototoxic insults have become a persistent target pursued for this hybrid system. In this study, we developed a composite GelMA/PEDOT:PSS conductive hydrogel that is suitable as a coating for the cochlear implant electrode for the potential local delivery of otoregenerative and otoprotective drugs. Various material characterization methods (e.g., ^1^H NMR spectroscopy, FT-IR, EIS, and SEM), experimental models (e.g., murine cochlear organoid and aminoglycoside-induced ototoxic HEI-OC1 cellular model), and biological analyses (e.g., confocal laser scanning microscopy, real time qPCR, flow cytometry, and bioinformatic sequencing) were used. The results demonstrated decent material properties of the hydrogel, such as mechanical (e.g., high tensile stress and Young’s modulus), electrochemical (e.g., low impedance and high conductivity), biocompatibility (e.g., satisfactory cochlear cell interaction and free of systemic toxicity), and biosafety (e.g., minimal hemolysis and cell death) features. In addition, the CDR medicinal cocktail sustainably released by the hydrogel not only promoted the expansion of the cochlear stem cells but also boosted the trans-differentiation from cochlear supporting cells into hair cells. Furthermore, hydrogel-based drug delivery protected the hair cells from oxidative stress and various forms of programmed cell death (e.g., apoptosis and ferroptosis). Finally, using large-scale sequencing, we enriched a complex network of signaling pathways that are potentially downstream to various metabolic processes and abundant metabolites. In conclusion, we present a conductive hydrogel-based local delivery of bifunctional drug cocktails, thereby serving as a potential solution to intracochlear therapy of bimodal auditory rehabilitation and diseases beyond.

## 1. Introduction

### 1.1. Cochlear Implantation and Electroacoustic Stimulation

Hearing loss is the fourth highest cause of disability globally, affecting over 450 million people, with an estimated annual cost of over 750 billion dollars [1]. Currently, no FDA-approved pharmacotherapy or surgery can reverse sensorineural hearing loss (SNHL) and reinstate normal acoustic hearing [2]. Therefore, contemporary clinical practice aims for the prevention of hearing loss and rehabilitation using hearing aids (HAs) or cochlear implants (CIs). Since the initial practical development of the CI 50 years ago [3], the indication of CI surgery has been bilateral profound SNHL with poor performance in speech audiometry despite help by HAs [4].

**Figure 1:**
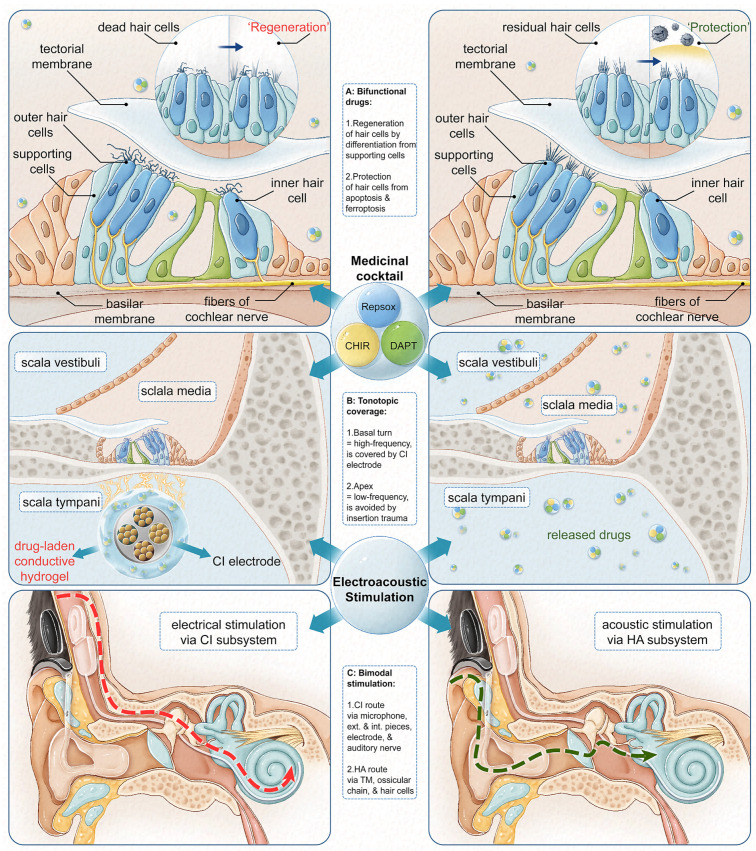
The potential clinical scenario behind this proof-of-concept cellular study: bimodal stimulation used in the cochlear implant–hearing aid hybrid system, tonotopic coverage by conductive hydrogel-coated CI electrode and intracochlear drug delivery, and bifunctional medicinal cocktail used for cochlear hair cells generation and protection.

Continuous technological, medical, and surgical innovations have broadened the indications of this interdisciplinary, state-of-the-art, otologic therapy. For example, in 2014, a hybrid CI–HA system employing electroacoustic stimulation (EAS) was first fitted for patients with intact low-frequency hearing and profound ski-sloped high-frequency hearing loss [5] (Figure 1C). The use of a specialized CI electrode reduces apical cochlear trauma and facilitates the preservation of pre-operative low-frequency hearing [6]. The EAS system provides additional advantages such as enhanced speech and pitch perception, hearing in background noise, and music appreciation [7]. However, this hybrid device is also facing considerable challenges [8]. 

### 1.2. Pharmacotherapy for Cochlear Hair Cell Generation and Protection

One of the biggest challenges in CI care is the post-implantation preservation of residual hearing [9]. Iatrogenic trauma often triggers an inflammatory cascade in which both acute insertion injury and chronic foreign body reaction can cause intracochlear fibrosis and the progressive loss of residual hearing [10]. Since hearing preservation has been found to be positively related with improved speech perception in CI patients [11], various strategies have been explored, such as gentle surgical technique, soft electrode design, and pharmacotherapy. However, only limited options of medication have been trialed, with corticosteroids being the most extensively studied drug because of its well-established anti-inflammatory effect on cochlear tissue [12]. 

In addition to corticosteroids, few other medications have also demonstrated otoprotective effects although with much less evidence documented, such as JNK inhibitors, TNF inhibitors, growth factors, and neurotrophin [12]. However, none of the medications mentioned above can restore dead cochlear hair cells. Recent advances in identifying small molecules that can induce the expansion and differentiation of cochlear supporting cells into sensory hair cells have offered a potential avenue for hair cell restoration [13]. Nonetheless, whether the small-molecule and growth factor cocktails used in this study also possess a hair cell-protective effect has not been confirmed. 

### 1.3. Inner Ear Drug Delivery Using Biomaterial-Modified Cochlear Implant Electrode 

Drug delivery to the inner ear was quite challenging until the advent of CI. In the pre-CI era, medications were administered either systemically or via the intratympanic route, with each posing specific disadvantages. Systemic delivery not only carries risks of adverse effect in vital organs but also suffers from unsatisfactory intracochlear drug concentration caused by the first pass effect and blood–labyrinth barrier [14]. On the other hand, intratympanic delivery relies on slow, passive, and incomplete diffusion through the round window membrane, which causes inconsistent and unpredictable drug concentrations in the perilymph [15]. 

The use of biomaterials has achieved CI electrode-based intracochlear drug delivery into the scala tympani [16] (Figure 1B). The unique advantages of this system include, but are not limited to, higher peak concentrations, prolonged drug delivery, and a minimal basal-to-apical drug gradient [17]. Various categories of biomaterial have been used as electrode coatings for this purpose, such as hydrogels, nanofibers, carbon nanotubes, and conductive polymers [18]. Emerging composites of these materials, such as conductive hydrogel, have drawn significant attention due to their desirable mechanical properties (e.g., soft and elastic), electrical properties (e.g., decent charge transfer), and biological properties (e.g., mimicking ECM) [19,20]. 

### 1.4. Conductive HG-Facilitated Generation and Protection of Hair Cells

In this in vitro study, we developed a conductive hydrogel using gelatin methacrylate (GelMA) and poly(3,4-ethylenedioxythiophene) (PEDOT) for the delivery of a bifunctional cocktail (i.e., CHIR, RepSox, and DAPT) by exerting an ‘otoregenerative’ effect via the differentiation of cochlear supporting cells to hair cells and an ‘otoprotective’ effect via the protection of hair cells from destructive stimuli in the cochlea (Figure 1A). Our strategy has a strong implication for bimodal listening used by the EAS hybrid CI–HA system, as it might potentially produce functional hair cells for mid- and high-frequency stimulation while maintaining residual hair cells, which are responsible for low-frequency stimulation in a partially deaf cochlea. This is supported by the findings from Gifford et al., in which the spectral overlap of acoustic and electric stimuli could provide better speech understanding and less listening effort in a noisy setting [21]. Furthermore, the rationale behind our bifunctional system for bimodal listening might be transferred and applied in other auditory implants and neural prosthetics used in the central nervous system (CNS).

## 2. Materials and Methods

### 2.1. Preparation of the Hydrogel

GelMA was synthesized with approximately 80% methacrylation (Figure S1). First, 10 g of type A porcine skin gelatin (Sigma-Aldrich, Beijing, China) was dissolved in 100 mL of pH 7.8 phosphate-buffered solution (PBS) (Shanghai Yuanye Biotech Co., Shanghai, China) at 60 °C. A volume of 10 mL of methacrylic acid anhydride (Sigma-Aldrich) was added during stirring. After 3 h, 300 mL of PBS was introduced and allowed to react for 1 h before quenching. The solution was then dialyzed against deionized water (refreshed twice daily) using a dialysis bag at 40 °C with a molecular weight cut-off (MWCO) of 12–14 kDa for 5 days to remove unreacted methacrylic anhydride. Finally, the GelMA product underwent freeze-drying process for 4 days to obtain fibrous white foam.

The composite hydrogel was prepared as follows. The GelMA prepolymer was dissolved in deionized water or PBS (pH 7.2–7.4, Shanghai Macklin Biochemical Technology Co., Shanghai, China) until a final concentration of 10% (*w*/*v*). To ensure uniform mixing, 1% (*w*/*v*) photoinitiator I2959 (Shanghai Titan Co., Shanghai, China) was added and stirred in the dark for 30 min. The commercially available PEDOT:PSS solution (Aladdin Scientific Corp., Shanghai, China) was subjected to sterile filtration (0.22 μm) to eliminate large aggregates. Subsequently, PEDOT:PSS was added to the GelMA solution, resulting in 0%, 0.1%, and 0.3% of the final concentration. The precursor solution was then injected into a reaction cell, and irradiated with 365 nm ultraviolet light for a total duration of 90 min (45 min exposure, flipped over, and another 45 min exposure) using a 100 w light curing machine (GH03-4B, Suzhou SoundLink Co., Suzhou, China).

### 2.2. Verification of Hydrogel Production

To confirm successful preparation of GelMA and quantify methacrylation, gelatin and GelMA were dissolved in DMSO-d_6_ (Shanghai Meryer Biochemical Technology Co., Shanghai, China) at 40 °C, and analyzed using nuclear magnetic resonance hydrogen (^1^H NMR) spectroscopy (Ultrashield, Bruker, Shanghai, China). The ^1^H resonance frequency used was set at 400 mHz. The degree of methacrylation or degree of substitution (DS) was defined as the ratio of the number of methacrylic groups present on gelatin to the total number of amino groups (e.g., lysine and hydroxylysine) in the gelatin prior to the reaction (DS(%) = (1 − P2/P1) × 100). In this analysis, the P2 and P1 represented the peak areas at 3.1 and 2.9 ppm for the proton peaks of lysine methylene in gelatin and GelMA, respectively.

To confirm successful preparation of the GelMA/PEDOT:PSS composite hydrogel, infrared absorption spectra were obtained for pure GelMA, pure PEDOT:PSS, GelMA/0.1% PEDOT:PSS, and GelMA/0.3% PEDOT:PSS using Fourier-transform infrared spectroscopy (FT-IR) (Nicolet iS50, ThermoFisher Scientific, Shanghai, China).

### 2.3. Mechanical Analysis of the Conductive Hydrogel

**Figure 2:**
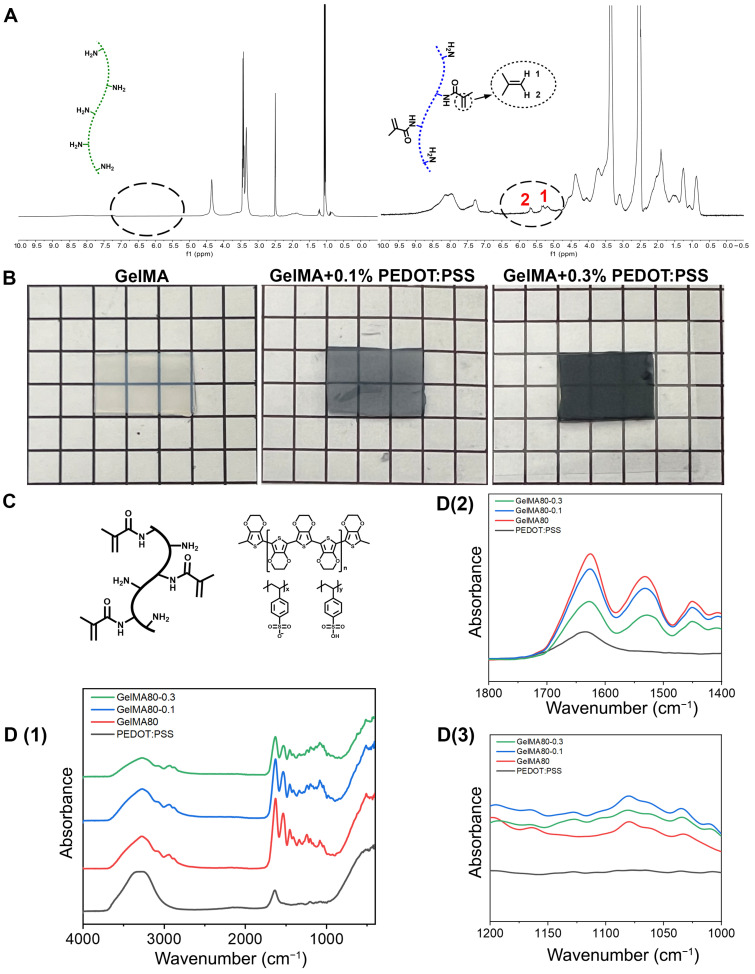
Fabrication and verification of the GelMA/PEDOT:PSS conductive hydrogel. (**A**) Degree of substitution of methacrylic acid on gelatin was determined using ^1^H NMR spectroscopy. (**B**) Macroscopic morphology of pure GelMA and composite hydrogels. (**C**) Chemical composition of GelMA and PEDOT:PSS. (**D**) The infrared spectra of GelMA and composite hydrogel by FT-IR. (**D1**–**D3**) are sequential enlargement of wavelengths of interest.

The polymerized sample was carefully cut into 15 mm × 5 mm × 1 mm pieces and subjected to tensile testing using an electromechanical universal testing machine (UTM2103, Shenzhen SUNS Technology Stock, Co., Shenzhen, China). The test was conducted at a controlled rate of 30 mm/min until fracture occurred. For the determination of mechanical properties, the Young’s modulus of elasticity was calculated as the slope of the linear portion of the strain ranging from 5% to 15% (Figure 2A). The breaking tensile strain was identified as the point at which the hydrogel sample fractured.

### 2.4. Electrochemical Analysis of the Conductive Hydrogel

Electrochemical impedance spectroscopy (EIS) was conducted at room temperature using an advanced electrochemical workstation (CHI760E, Shanghai CH Instruments, Shanghai, China). A simple yet effective four-electrode method was employed to measure the electrical resistivity of the material. To ensure optimal contact between the electrodes and the hydrogel, the sample was precisely cut into a rectangular piece measuring 30 mm × 5 mm × 1 mm, with the sample positioned between and perpendicular to the two parallel silver sheets. The conductivity was calculated as follows: σ = L/(Z × A).

### 2.5. Morphological Analysis of the Conductive Hydrogel

The impact of PEDOT:PSS on the surface morphology of GelMA hydrogel was investigated using scanning electron microscopy (SEM). The sample was appropriately sized, quenched in liquid nitrogen, and subsequently freeze-dried (FreeZone 4.5l, Lanconco Corporation, Kansas City, MO, USA) for 5 days. Following freeze-drying, a layer of Pt/Pd alloy was sputtered and coated onto the sample, allowing for the observation of cross-sectional morphology and structure using a scanning electron microscope (Regulus 8230, Hitachi, Tokyo, Japan). Cells attached to the material surface were visualized according to previous protocols [22].

### 2.6. Calculation of Swelling Ratio of the Conductive Hydrogel

Rectangular pieces of hydrogel were prepared as described in Section 2.1. Dry weights of the hydrogel were recorded, and the samples were submerged in PBS and then placed into an incubator at standard culture conditions (37 °C, 5% CO_2_). Samples were removed and weighed at different time points (1, 2, 4, and 6 days). The swelling ratio was calculated as follows: (Wwet – Wdry)/Wdry × 100%, where Wdry is the original weight and Wwet is after removal from PBS.

### 2.7. Degradation and Stability Test of the Conductive Hydrogel

For the in vitro degradation test, rectangular samples were prepared. Dry weights of the hydrogel were measured at time point 0, and then samples were submerged in PBS supplemented with 10% fetal bovine serum (FBS) and incubated at 37 °C. The residual hydrogel was removed from the solution at different time points of 1, 2, 3, 4, and 6 weeks, when they were rinsed with DI water, and the degraded dry weight was recorded. A similar experiment was conducted using collagenase to simulate enzymatic degradation at a collagenase concentration of 0.4 mg/mL in DMEM at 37 °C. For the in vivo degradation test, the conductive hydrogel samples were implanted subcutaneously at the dorsum of C57BL/6 mice. 

### 2.8. Drug Loading in and Release from the Conductive Hydrogel

The key drug cocktail used for hair cell regeneration and protection in our study included CHIR (S1263), RepSox (S7223), and DAPT (S2215) from Selleck Chemicals, Houston, TX, USA. During hydrogel preparation (Section 2.1), these medications were added in PBS before crosslinking (the respective combinations for proliferation and differentiation were explained in Section 2.11). During hydrogel degradation in a physiological solution, the profile of drug release from the hydrogel was examined using dexamethasone as a model drug. The supernatant of the conductive hydrogel was collected and measured using a micro-UV-vis spectrometer (NanoDrop™ One, Thermo Scientific, Waltham, MA, USA) against a calibration curve to quantify the released drug at 1, 2, 4, and 6 days. 

### 2.9. Analysis of Biocompatibility and Biosafety of the Conductive Hydrogel

The in vitro biocompatibility of the hydrogels, such as polyacrylamide (PAM) (control), GelMA, and composite hydrogels, was assessed using the House Ear Institute-Organ of Corti 1 (HEI-OC1) cells. The biocompatibility was determined based on the following parameters: cell viability (Section 2.14), apoptosis (Section 2.15), and light microscopic morphology and scanning electron microscopic morphology (Section 2.5). The in vivo biocompatibility of the hydrogels was assessed using various histological staining methods such as hematoxylin and eosin (H&E), Masson and Hoechst staining. Tissues and hydrogel samples were fixed in formalin and embedded in paraffin, sectioned, and stained. 

The in vitro biosafety was analyzed using the hemolysis test [23]. In brief, biomaterials were incubated in triplicates with diluted blood for at least 3 h at 37 °C. After centrifugation, the plasma free hemoglobin concentration was measured using spectrophotometric detection at 540 nm. The in vivo biosafety of the conductive hydrogel samples implanted subcutaneously at the dorsum of C57BL/6 mice was determined by assessing systemic toxicity in various vital organs such as heart, liver, spleen, lung, and kidney using H&E staining.

### 2.10. Animals Strains Used for Various Studies

All animal studies were conducted under an approved institutional protocol according to National Institutes of Health guidelines. For biocompatibility analysis, female C57BL/6 mice at 8 weeks of age were obtained from Vital River Laboratory Animal Technology Co., Ltd., China. To observe the proliferation of Lgr5^+^ supporting cells, Lgr5-EGFP-IRES-Cre-ER mice (The Jackson Laboratory, strain 8875) [24] were used to analyze the effects of small molecules on cochlear stem cell expansion. The same mice were crossed with Rosa26-td-Tomato reporter mice (The Jackson Laboratory, strain 7909) [25] to create a mouse line that enabled lineage tracing of the cells that resulted from differentiated Lgr5-expressing cells. To observe Atoh-1-expressing hair cells, Atoh1-nGFP mice [26] were used to identify differentiated hair cells. 

### 2.11. Separation and Differentiation of Cochlear Supporting Cells

A detailed protocol for the isolation and differentiation of cochlear supporting cells can be found in the previous paper [13]. In brief, the cochleae of neonatal mice were dissected, the organ of Corti was separated from the stria vascularis and the modiolus, and the cochlear epithelium was separated from the underlying mesenchyme. Single cells obtained from the epithelia were suspended in a Matrigel (Corning, New York, NY, USA) dome for 3D culture.

To support proliferation of supporting cells, cells were cultured in a 3D system and bathed in advanced Dulbecco’s modified Eagle’s medium (DMEM)/F12 (Gibco, Shanghai, China), supplemented with Glutamax (Gibco), N2, B27 (Gibco), growth factors, and small molecules released from the hydrogel, including CHIR (3 μM) and RepSox (1.5 μM). Media were changed every other day. To support the differentiation of stem cell colonies, both the expansion media and expansion hydrogel were removed, and colonies remained in 3D culture. Same basal and supplemental media were used, with the addition of CHIR (3 μM) and DAPT (3.75 μM) released from the hydrogel. The optimal drug concentration used above was determined by analyzing a gradient of loading and released drug concentrations from hydrogel-based drug delivery system (Figure S2).

### 2.12. Immunofluorescent Microscopy for Cochlear Cells in an Organoid

The cochlear organoids were fixed at room temperature in 4% paraformaldehyde/PBS for 10 min and then washed in PBS. Permeabilization of the cellular membrane (0.3% Triton X-100) was followed by blocking solution (15% heat inactivated goat serum in PBS for 1 h). Diluted primary antibodies were applied overnight at 4 °C. Secondary antibodies (Alexa 488, Alexa Fluor 568, and Alexa Fluor 647 conjugated; Invitrogen, Waltham, MA, USA) were used at 1:500 dilutions [13]. Nuclei were visualized with DAPI (Vector Laboratories, Newark, CA, USA).

### 2.13. Cell Culture of HEI-OC1 Cochlear Cells

The House Ear Institute-Organ of Corti 1 (HEI-OC1) cells were sourced from House Ear Institute (Los Angeles, CA, USA) and cultured in high-glucose DMEM containing 10% FBS in a humidified atmosphere of 10% CO_2_ at 33 °C, which is a permissive condition for this cell line [27]. Common cell-culture-based antibacterial cocktails containing penicillin or streptomycin were avoided. The exact culture conditions for various cellular experiments, such as cell proliferation, viability, and programmed cell death followed previously published protocols [28]. 

### 2.14. Establishment of an Aminoglycoside-Induced Ototoxic Model

To establish a model of ototoxic medication-induced damage, a gradient concentration of neomycin (TargetMol Chemicals Inc., Shanghai, China) (e.g., 1, 2, 4, 6, 8 and 10 mM) was used on the HEI-OC1 cells (Figure S5). The incubation time was set at 24 h for all therapeutic experiments except for the oxidative stress analysis, in which 6 h was used. For the otoprotective studies, a medicinal cocktail containing CHIR (3 μM), RepSox (1.5 μM) and DAPT (3.75 μM), aka. CDR, released from the conductive hydrogel was used. 

### 2.15. Assessment of Cell Viability of HEI-OC1

The cells were inoculated into 96-well plates at 5000 cells per well, damaged by neomycin and rescued by drug-releasing hydrogel, and cultured for 24 h. Cell viability was measured using a Cell Counting KIT-8 (CCK-8) assay kit. The absorbance at 450 nm was obtained using a microplate reader (Infinite F50, Tecan, Morrisville, NC, USA). In addition, the Live/Dead assay was conducted to further verify the cell viability. In detail, cells were stained with Calcein AM (live) or ethidium homodimer-1 (dead) and incubated for 15 min. After staining, cells were washed with PBS and observed using a fluorescence microscope (CKX53, Olympus, Tokyo, Japan).

### 2.16. Measurement of Apoptosis in HEI-OC1 Cells

The HEI-OC1 cells were seeded into 6-well plates at a density of 2 × 10^5^ cells/well overnight and exposed to different concentrations of neomycin and therapeutic drug cocktails. The apoptosis was identified by flow cytometry using a PE Annexin V apoptosis detection kit I (BD Biosciences, San Jose, CA, USA) according to manufacturer’s instructions. In brief, the cells were collected, washed, resuspended in PBS, and incubated at 25 °C with PE Annexin V and 7-AAD for 15 min in the dark. The cells were then analyzed using a flow cytometer (BD LSRFortessa, BD Biosciences, USA) within 1 h. In addition to the quantitative flow cytometric analysis, the qualitative acridine orange and ethidium bromide (AO/EB) cell staining was used. The HEI-OC1 cells were stained with 2 μL AO and EB for 20 min at 37 °C. After that, cells were washed with PBS and detected using a fluorescence microscope.

### 2.17. RNA Isolation and Quantitative Real-Time PCR

The total RNA of HEI-OC1 cells and primary cochlear cells were extracted using the RNA-Quick Purification kit (RN001, Shanghai Yishan Biotechnology Co., Shanghai, China). The HiScript 1st Strand cDNA Synthesis Kit was used for reverse transcription PCR (RT-PCR). Quantitative real-time PCR (Q-PCR) was performed using the SYBR Green RT-qPCR Kit. All the above reagents and kits were purchased from Vazyme, Nanjing, China. The primers are listed in Supplementary Table S1. The Ct values were calculated using the ΔΔCt method, and the relative changes in mRNA levels were obtained by normalization to the glyceraldehyde phosphate dehydrogenase gene (GAPDH).

### 2.18. Quantification of Oxidative Stress in HEI-OC1 Cells

To evaluate intracellular levels of oxidative stress in the HEI-OC1 cells, the reactive oxygen species (ROS) levels were quantified using a Reactive Oxygen Species Assay Kit (S0033S, Beyotime Biotechnology, Shanghai, China) based on the detection of the fluorescence intensity of 2′,7′-dichlorofluorescein diacetate (DCFH-DA). After treatment by drugs released from the hydrogel, the cells were incubated with DCFH-DA (2 μM) for 15 min in the dark, collected, washed, and resuspended in PBS, and analyzed using a FACS Calibur system (BD Biosciences).

### 2.19. Measurement of Ferroptosis in HEI-OC1

Various nodes during ferroptosis were analyzed using different methods. Firstly, intracellular Fe^2+^ was detected using the Iron Colorimetric Assay Kit (BioVision, Milpitas, CA, USA). In brief, cells were homogenized in 500 µL iron assay buffer and centrifuged at 16,000× *g* for 10 min. After incubating the plate for 30 min at 37 °C, a 100 µL iron probe was added to each well and incubated for 60 min at 37 °C. The absorbance at 593 nm was measured using a microplate reader against a standard curve. In addition, Fe^2+^ within the HEI-OC1 cells were further verified using the Intracellular Iron Measurement FerroOrange fluorescent probe that selectively binds iron ions (Dojindo Laboratories, Kumamoto, Japan). Secondly, the lipid peroxide within the cells was measured using the Liperfluo fluorescent probe, which reduces lipid hydroperoxide to their hydroxy homologues to yield a fluorescent product (Dojindo). 

### 2.20. Transcriptomic Sequencing and Bioinformatic Analysis

The RNA sequencing (RNA-seq) was performed to detect the mRNA expression profiles of the control group, neomycin-exposed cells, and drug-cocktail-treated cells using the HiSeq3000 System (Illumina, San Diego, CA, USA). The LifeScope Genomic Analysis Software v2.5.1 was used to align the reads to the genome, generate raw counts corresponding to each known gene, and calculate the fragments per kilobase million (FPKM) values. First, the differentially expressed genes (DEGs), |logFC| ≧ 1, were clustered. Then, Gene Ontology (GO) and Kyoto Encyclopedia of Genes and Genomes (KEGG) analyses were performed using the R software (version 4.1.3).

### 2.21. Statistical Analysis

Statistical analyses were completed using GraphPad Prism (version 9). Comparisons between the continuous variable of subject groups were made by two-tailed Student’s *t*-test. ANOVA and Tukey’s post hoc tests were used to compare multiple conditions. The statistical significance was determined as *p* < 0.05. At least three repetitions of each experiment have been completed.

## 3. Results

### 3.1. GelMA/PEDOT:PSS Conductive Hydrogel Exhibited Desirable Material Properties

#### 3.1.1. GelMA/PEDOT:PSS Composite Hydrogel Was Successfully Prepared

The successful fabrication of the GelMA hydrogel was confirmed, and the degree of the substitution of methacrylic acid on gelatin was determined using ^1^H NMR spectroscopy. In the spectra of gelatin and GelMA, the lysine methylene signal appeared in the range of 2.9 to 3.1 ppm. In this analysis, the P2 and P1 represented the peak areas at 3.1 and 2.9 ppm for the proton peaks of lysine methylene in gelatin and GelMA, respectively (Figure 2A). In comparison, the peaks at 5.35 ppm and 5.68 ppm corresponded to the vibration of the C=C bond after methacrylation. This observation confirmed the successful modification of gelatin with MA. By integrating the peak area at 3.1 and 2.9 ppm, the degree of MA substitution on gelatin was estimated to be approximately 80% based on the formula (Section 2.2).

The successful preparation of the GelMA/PEDOT:PSS composite hydrogel was confirmed using FT-IR. In the infrared absorption spectra (Figure 2D), the pure GelMA exhibited characteristic peaks at wavenumbers of 1626 cm^−1^ which corresponded to the C=O bond vibration of the amino acid side chain, as well as 1530 cm^−1^ and 1240 cm^−1^, which corresponded to the stretching vibration of the N-H bond. Secondly, the pure PEDOT displayed characteristic peaks at wavenumbers of 839 cm^−1^, which represented the thiophene C-S-C bond; 1035 cm^−1^, which represented the ethylenedioxy C-O-C bond; and 1633 cm^−1^, which represented the C=C bond in the thiophene ring. Thirdly, the PSS exhibited characteristic peaks at wavenumbers of 780 cm^−1^, which corresponded to the vibration absorption of C-S bond, as well as 1008 cm^−1^ and 1175 cm^−1^, which corresponded to the -SO_3_ vibrations. Lastly, the GelMA/PEDOT:PSS composite hydrogel displayed all the aforementioned characteristic peaks, suggesting the successful preparation of this conductive hydrogel.

#### 3.1.2. GelMA/PEDOT:PSS Hydrogel Exhibited Satisfactory Mechanical Features

**Figure 3:**
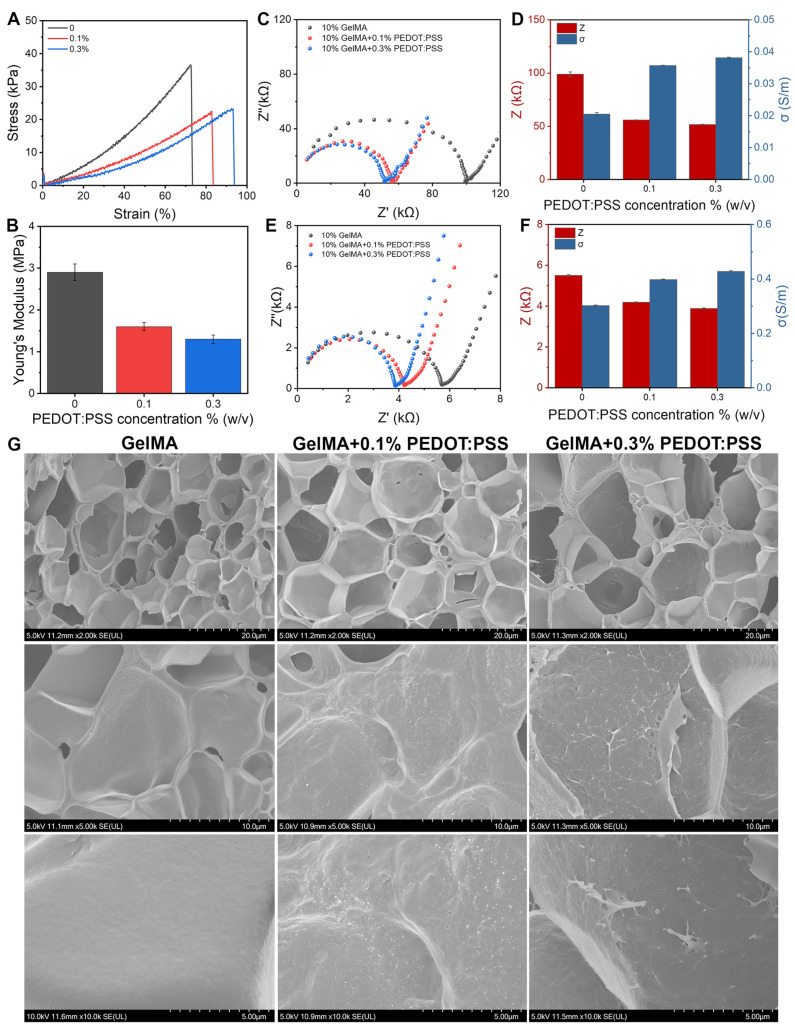
Material properties of the GelMA/PEDOT:PSS conductive hydrogel. (**A**,**B**) Mechanical characteristics. (**C**,**D**) Electrochemical characteristics when prepared using DI water. (**E**,**F**) Electrochemical characteristics when prepared using PBS. (**G**) Microscopic morphology of various hydrogels using SEM.

As the PEDOT:PSS concentration increased from 0% to 0.3%, the tensile stress of the GelMA hydrogel decreased from 23.6 kPa to 22.4 kPa (Figure 3A), whereas its Young’s modulus decreased from 2.9 ± 0.2 MPa to 1.6 ± 0.1 MPa and 1.3 ± 0.1 MPa (Figure 3B). The relatively high tensile stress and Young’s modulus observed in the GelMA hydrogel could be attributed to the utilization of a 10% (*w*/*v*) GelMA concentration during the sample preparation as higher solute concentrations tend to enhance the stress and modulus of the hydrogel [29]. The 7% GelMA hydrogel showed worse mechanical features, which made the manipulation of the samples in routine laboratory practice very difficult (Supplementary Videos S1 and S2). On the other hand, in comparison to the pure GelMA hydrogel, the inclusion of 0.1% and 0.3% PEDOT:PSS in the composite system exhibited a notable increase in the tensile strain at break. This improvement could be attributed to the electrostatic interaction between the PEDOT:PSS and GelMA, which lead to the formation of more electrostatic binding and the creation of a more flexible and stretchable network within the composite structure [29].

In the absence of PEDOT:PSS, pure GelMA appeared colorless and translucent, with efficient UV curing properties at 365 nm (Figure 2B). However, with the addition of PEDOT:PSS, the color of the hydrogel changed to a gray semi-transparent shade. This alteration in color indicated a decrease in the UV light penetration efficiency, which subsequently lead to a reduction in the degree of polymerization and, consequently, a decrease in the mechanical properties of the hydrogel.

#### 3.1.3. GelMA/PEDOT:PSS Hydrogel Exhibited Satisfactory Electrochemical Features

Firstly, the GelMA/PEDOT:PSS hydrogel was synthesized employing deionized water as the solvent. The composite hydrogel, comprising 0.1% and 0.3% PEDOT:PSS, exhibited a lower impedance compared to the pure GelMA hydrogel. Specifically, the impedance decreased from 99.0 kOhm for the pure GelMA hydrogel to 55.0 kOhm and 51.8 kOhm for the hydrogels containing 0.1% and 0.3% PEDOT:PSS, respectively (Figure 3D). Similarly, the conductivity of the hydrogels increased from 0.021 S/m for the pure GelMA hydrogel to 0.035 S/m and 0.038 S/m for the hydrogels containing 0.1% and 0.3% PEDOT:PSS, respectively. These findings suggested that the GelMA/PEDOT:PSS composite hydrogel possessed significantly enhanced conductivity.

Secondly, to simulate a physiological environment, the GelMA/PEDOT: PSS hydrogel was further prepared utilizing PBS solution with a pH range of 7.2–7.4. Surprisingly, replacing the DI water with PBS during hydrogel fabrication changed the electrochemical performance by at least 10 times. The impedance decreased from 5.5 kOhm for the pure GelMA hydrogel to 4.2 kOhm and 3.9 kOhm for the hydrogels containing 0.1% and 0.3% PEDOT:PSS, respectively, whereas the conductivity of the hydrogels increased from 0.30 S/m for the pure GelMA hydrogel to 0.40 S/m and 0.43 S/m for the hydrogels containing 0.1% and 0.3% PEDOT:PSS, respectively (Figure 3F). The achieved conductivity range of 0.40–0.43 S/m for the GelMA/PEDOT:PSS hydrogel prepared using PBS solution surpasses the conductivity of artificial perilymph, which is 0.186 S/m [30]. Our conductivity range satisfies the requirements for effectively transmitting bioelectrical signals from the CI electrode to the cochlea microenvironment, thereby facilitating cochlear cell proliferation and differentiation.

#### 3.1.4. GelMA/PEDOT:PSS Hydrogel Displayed a Cell-Friendly 3D Porous Morphology

The SEM images depicted in Figure 3G illustrated the cross-sectional morphology of both the pure GelMA and GelMA/PEDOT:PSS composite hydrogels. Firstly, it revealed characteristic microporous structures for all the hydrogel samples. The porous structure within a biomaterial could facilitate drug release and 3D cell growth, which are two pre-requisites for our study [31]. Secondly, the pure GelMA hydrogel exhibited a relatively smooth pore wall without the presence of particles. However, the GelMA/PEDOT:PSS composite hydrogel displayed particles of varying sizes and distributions, which were either embedded or adsorbed onto the hydrogel’s pore wall (Figure 3G). Thirdly, the composite hydrogel containing 0.1% PEDOT:PSS showcased an even distribution of particles with similar sizes on its surface. The particle size was relatively uniform, with a diameter ranging from approximately 50 to 100 nm. In comparison, the surface of the composite hydrogel containing 0.3% PEDOT:PSS not only contained small-sized point particles with a diameter of approximately 50–100 nm, but also larger particles with a diameter of around 200–400 nm. The formation of these larger particles could be attributed to the increased concentration of PEDOT:PSS, which tends to result in uneven dispersion and the formation of larger aggregates. 

### 3.2. Conductive Hydrogel Shows Reasonable Biostability and Biodegradation

**Figure 4:**
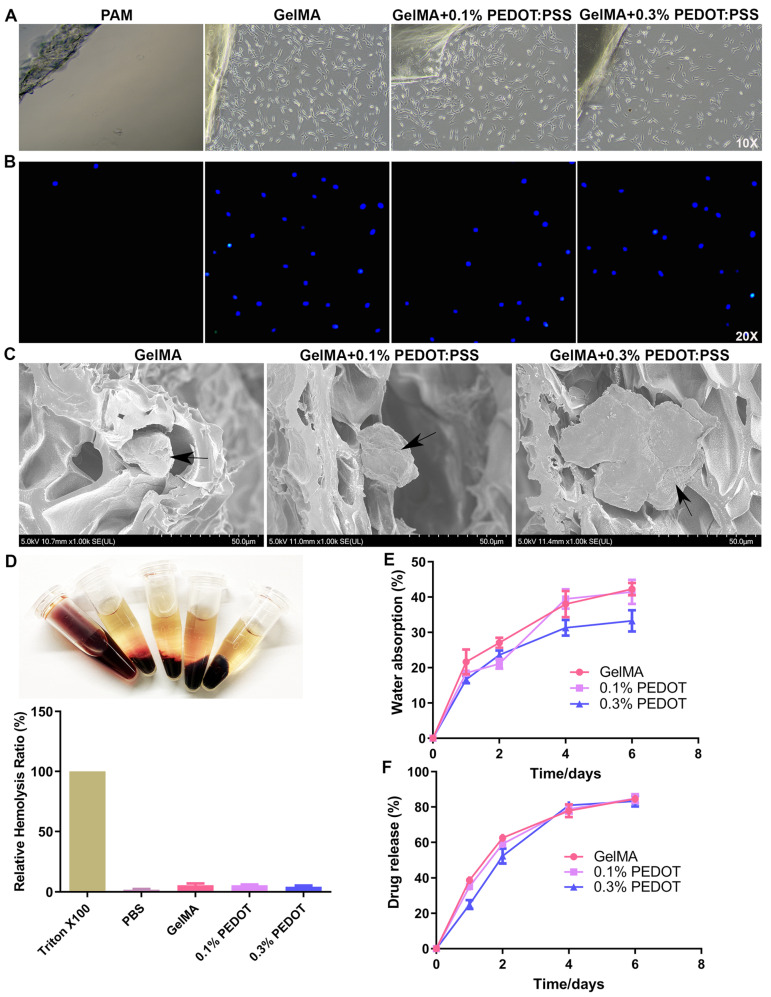
Biocompatibility, biosafety, and drug release of the GelMA/PEDOT:PSS conductive hydrogel. (**A**) Biomaterial–cell interaction observed by light microscopy. (**B**) Biomaterial–cell interaction observed by fluorescent microscopy with DAPI nuclei staining. (**C**) Hydrogel–cell interaction observed by SEM. Black arrows indicate HEI-CO1 cells attached on the porous structure of hydrogel. (**D**) Hemolysis test to simulate blood–material contact during surgical practice. (**E**) Swelling ratio of various hydrogels. (**F**) Drug release from the conductive hydrogel.

Hydrogels can swell in aqueous solution and retain a large fraction of water without dissolving. The swelling capacity of a hydrogel is of great importance as medications can be loaded into hydrogel to achieve controlled drug release [32]. The profile of the water absorption versus time of a hydrogel sample in PBS was obtained by performing free-absorbency capacity measurements at consecutive time intervals (Section 2.6). Our results showed that the swelling ratio of all the hydrogels plateaued around day 4 of submersion (Figure 4E). The water absorption profile of the GelMA hydrogel reached 42% at day 6. However, the swelling ratio of the hydrogels decreased with an increasing content of PEDOT:PSS (from 39% for 0.1% PEDOT to 33% for 0.3% PEDOT). A plausible explanation for the lower swelling ratio for the composite hydrogel could be dampened light penetration in the sample or the cross-reactivity of the radical from PEDOT, causing the un-crosslinked GelMA to diffuse from the sample [29].

On the other hand, the results of the in vitro hydrolytic and enzymatic degradation and in vivo biological degradation demonstrated a similar pattern, in which the weight of the hydrogel decreased steadily with a similar rate among all three hydrogels until day 3 (Figure S2B,C). After that, the degradation of the 0.3% PEDOT composite hydrogel decelerated more significantly compared to the other two hydrogels. The hybrid hydrogel seemed more stable, which is most likely caused by the more abundant synthetic PEDOT:PSS component [33]. The main difference between the in vitro and in vivo degradation of the hydrogel rested in the time required for complete decomposition (6 weeks vs. 8 weeks). The absence of macrophage infiltration in the composite hydrogel could also be the reason for the slower hydrogel degradation in vivo (Figure S2D).

### 3.3. Conductive Hydrogel Demonstrates Remarkable Biocompatibility and Biosafety

As illustrated in Figure 4A, the control polymeric hydrogel, polyacrylamide, showed very poor cytocompatibility as there were very little robust HEI-OC1 cells near it (Figure 4A and Figure S2A). In comparison, both the GelMA and composite hydrogels exhibited excellent biocompatibility. The hydrogels not only displayed a chemokine effect for the cochlear cells (Figure 4A) but also an intragel cellular growth (Figure 4B). In addition, these hydrogels acted as a 3D scaffold supporting cell attachment as visualized using SEM (Figure 4C). Being consistent with the in vitro results, the in vivo results demonstrated similar patterns (Figure S2D). The H&E staining revealed that cells can grow into the gel without causing severe inflammatory cell infiltration. The Masson staining failed to detect severe fibrosis around the gels. Once again, the Hoechst staining confirmed the intramaterial growth of the various cells in situ (Figure S2D).

The biosafety of our hydrogels was determined according to their hemocompatibility, cell death, and systemic toxicity. The hemolysis test was conducted to simulate blood–material contact during surgical practice [34]. As shown in Figure 4D, the GelMA and composite hydrogels used in our study did not reveal any significant hemolysis. The cell death was analyzed using the Live/Dead assay and fluorescent AO/EB assay. As shown in Figure S3, the PAM control demonstrated considerable cell death and apoptosis. In contrast, both hydrogels showed much less programmed cell death. In addition, the decent biosafety demonstrated by our hydrogel samples did not seem to be cell line-specific, as both PC12 neuronal cells and HEI-OC1 cochlear cells displayed similar results, which has strong clinical implication as mammalian cochlea consists various cell types, e.g., hair cells and spiral ganglion neurons (SGNs) [35]. Finally, the systemic toxicity analysis did not reveal significant damage to various vital organs, such as heart, liver, spleen, lung, and kidney (Figure S6).

### 3.4. Conductive Hydrogel Supports Sustained Release of Medicinal Cocktail

The drug release profile of all three types of hydrogels was depicted using UV-vis spectrometry (Figure 4F). Firstly, all the samples demonstrated a similar release pattern, i.e., early burst release followed by a slower release until day 6, when approximately 80% of the drug depot was emptied. Secondly, the 0.3% PEDOT:PSS hydrogel exhibited a slower release profile in the initial period when compared to the pure GelMA and composite hydrogel containing 0.1% PEDOT:PSS. Lastly, this difference in drug release profile leveled out from day 4. The possible mechanism behind the results where increasing PEDOT content in the hydrogel slowed down the drug release could be caused by the electrostatic interactions between the drugs and conductive polymer or pores of hydrated hydrogel network [36]. 

### 3.5. Conductive Hydrogel Facilitates Cochlear Cell Expansion in Cochlear Organoids

**Figure 5:**
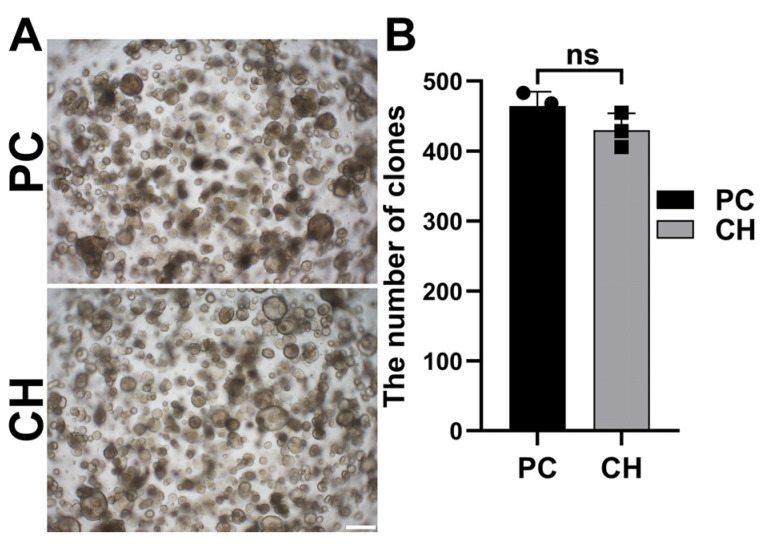
Organoid compatibility of the GelMA/PEDPT:PSS hydrogel. (**A**) Qualitative assessment by bright-field microscopy showing Atoh1-GFP colonies derived from inner ear epithelial cells. Scale bar = 200 µm. These colonies were obtained during a 7-day period of proliferation, then subjected to a 10-day culture period aimed at inducing cellular differentiation supplemented by EFIPVCDR (EGF, bFGF, IGF-1, pVc, VPA, CHIR, DAPT, and Repsox). For the negative control group, inner ear epithelial cells were cultured in the company of EFIPV without CDR. (**B**) Quantitative analysis of number of colonies from inner ear epithelia. n = 3 independent samples. PC = positive control. CH = conductive hydrogel. ns = not statistically significant.

Firstly, to further test the organoid compatibility of the GelMA/PEDPT:PSS hydrogel, we employed a previously established cochlear organoid model [13]. As evidenced by Figure 5A,B, the incorporation of the GelMA/PEDOT:PSS hydrogel in the organoid culture maintained comparable organoid-forming efficacy with the control groups. These results suggested that the conductive hydrogel is biocompatible to support the proliferation or the structural integrity of cochlear organoids, suggesting its potential compatibility in applications involving cochlear tissues.

Secondly, an optimal concentration of medicinal cocktail released by the GelMA/PEDPT:PSS hydrogel was identified. As the bright field images in Figure S4 clearly revealed, the positive control group resulted in a significantly higher efficiency in hair cell production when compared to the negative control group. Using the positive and negative control groups as a benchmark, a too low (2 μM) or too high concentration (4, 5, 6 and 10 μM) of the drug cocktail would cause suboptimal cochlear cell expansion. 

**Figure 6:**
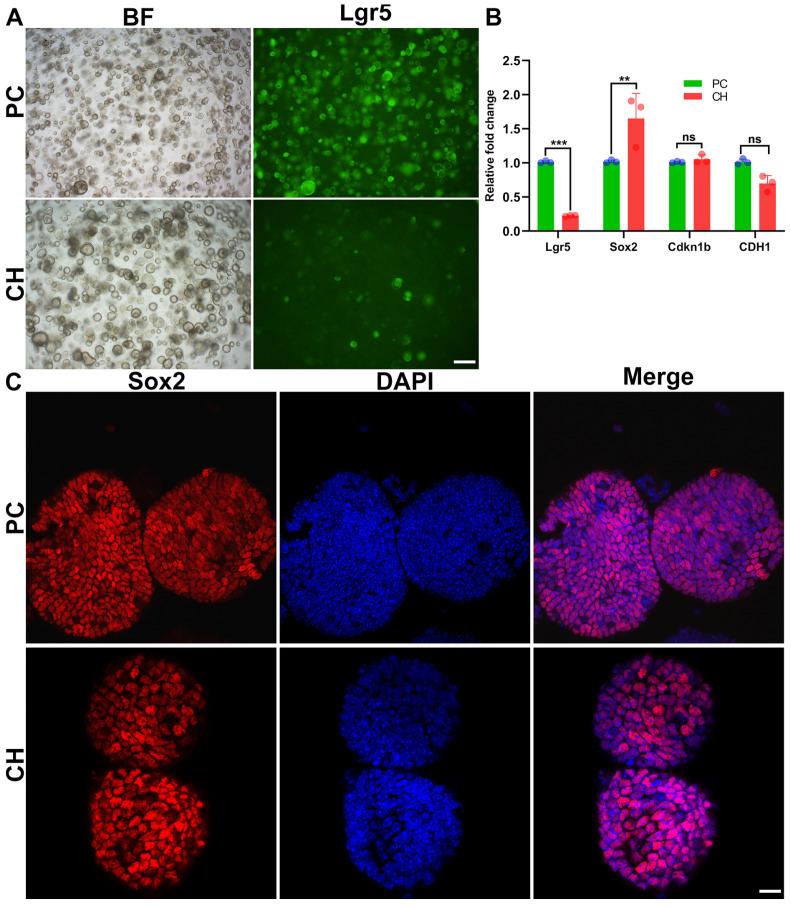
Cochlear cell expansion by conductive hydrogel-released drugs in a cochlear organoid. (**A**) GFP fluorescence and bright-field images of Lgr5-GFP colonies obtained from inner ear epithelial cells. Scale bar = 200 µm. (**B**) Essential cochlear stem cell markers (Lgr5, Sox2, Cdkn1b, and CDH1) were compared using real-time qPCR between the positive control and composite hydrogel groups. n = 3 independent samples per gene. Error bars represent mean ± SEM; *p* < 0.05 for all genes presented; ** *p* < 0.005, *** *p* < 0.001. (**C**) Colonies generated from inner ear epithelial cells expressed the cochlear progenitor cell marker Sox2 in their nuclei. Scale bar = 20 µm. PC = positive control. CH = conductive hydrogel. ns = not statistically significant.

Thirdly, the drug release from the GelMA/PEDPT:PSS hydrogel supported the expansion of the cochlear supporting cells. In order to ascertain whether our conductive hydrogel could maintain the stemness of the cochlear cells in organoids from a Lgr5-GFP mouse, fluorescent microscopy and qPCR were used. The results showed that the expression level of Lgr5, a stem cell marker [37], was reduced in the conductive hydrogel group (Figure 6A,B) compared to direct drug administration. Conversely, Sox2, which is another stem cell marker [38], showed higher expression in the conductive hydrogel group (Figure 6B,C). Additionally, other markers such as cyclin-dependent kinase inhibitor 1b (Cdkn1b) [39] and E-cadherin (CDH1) [40] were maintained (Figure 6C). 

### 3.6. Drug Cocktail Released by Conductive Hydrogel Promotes the Generation of Hair Cells 

**Figure 7:**
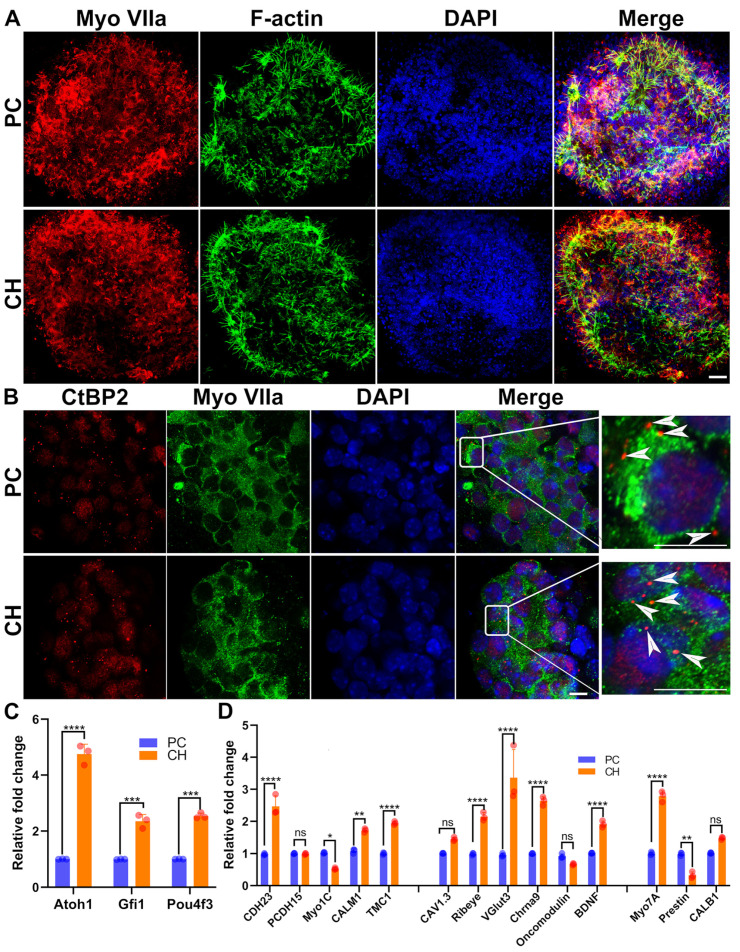
Cochlear cell generation by conductive hydrogel-released drugs in a cochlear organoid. (**A**) Myosin VIIa^+^ cells and F-actin-rich bundles on the cell surface both in the positive control and composite hydrogel groups. Scale bar = 20 μm; n = 3. (**B**) Myosin VIIa^+^ cells contained CtBP2^+^ ribbon synapse-like puncta at the basal end of the cell near the membrane (arrowheads). Scale bar = 20 μm; n = 3. (**C**) Key hair cell differentiation-related genes were compared using real-time qPCR between the positive control and composite hydrogel groups; n = 3 independent samples per gene. Error bars represent mean ± SEM. (**D**) Fourteen hair cell function-related genes were compared using real-time qPCR between the positive control and composite hydrogel groups. n = 3 independent samples per gene. Error bars represent mean ± SEM. * *p* < 0.01, ** *p* < 0.005, *** *p* < 0.001, **** *p* < 0.0001.

The crucial biomarkers related to hair cell differentiation (e.g., Myosin VIIa) and hair cell development and function (e.g., F-actin and CtBP2) were analyzed using immunofluorescence staining (Figure 7A,B). Among these three markers, only CtBP2 exhibited a significantly stronger staining intensity in the conductive hydrogel group, whereas the levels of myosin VIIa and F-actin were similar between the positive control and composite hydrogel groups. 

In order to further explore the hair cell-generative effect of the drugs released from the conductive hydrogel at a larger scale, qPCR analysis was conducted (Figure 7C,D). The expression of nearly twenty genes of interest were measured, including genes encoding transcriptional factors involved during hair cell differentiation (3 genes) and those responsible for hair cell structure and function (14 genes). On the one hand, all the hair cell differentiation-related genes, i.e., Atoh1, Gfi1, and Pou4f3, were upregulated in the conductive hydrogel group. On the other hand, hair cell function-related genes were further categorized and analyzed across three subsets: transduction (e.g., CDH23, PCDH15, Myo1C, CALM1, and TMC1), neurotransmitter (e.g., CAV1.3, Ribeye, VGlut3, Chrna9, Oncomodulin, and BDNF), and cell type classification (Myo7A, Prestin, and CALB1). Notably, 10 out of the 14 assessed genes demonstrated significant regulation, indicating a broad enhancement of hair cell function markers. For instance, genes involved in mechanoelectrical transduction, i.e., CDH23, CALM1, and TMC1, showed marked expression increases. Genes associated with calcium channel activity (CAV1.3), synaptic ribbon formation (Ribeye), vesicular glutamate transport (VGlut3), cholinergic receptor activity (Chrna9), calcium binding (Oncomodulin), and neurotrophic factor production (BDNF) were also upregulated.

### 3.7. Drug Cocktail Released by Conductive Hydrogel Protects Hair Cells from Apoptosis

**Figure 8:**
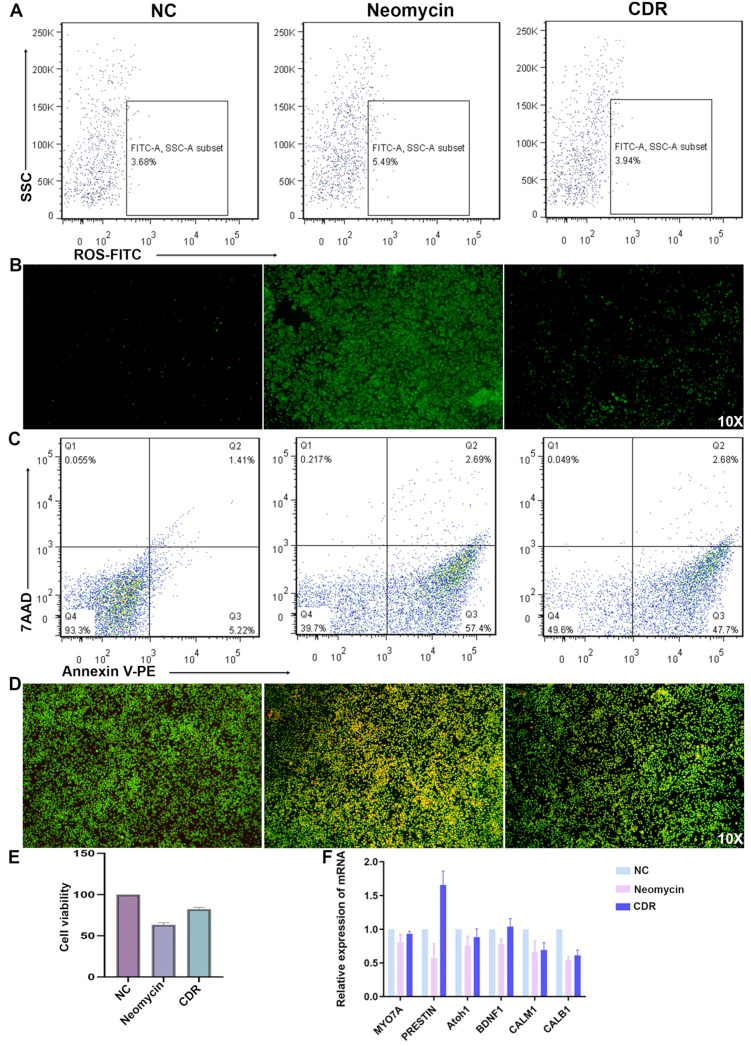
Protection of HEI-OC1 hair cells from apoptosis. (**A**,**B**) Flow cytometric analysis and fluorescent microscopic images of reactive oxygen species induced by neomycin and rescued by CDR drug cocktail. (**C**,**D**) Flow cytometric analysis and fluorescent microscopic images of cell apoptosis induced by neomycin and rescued by CDR drug cocktail. (**E**) Cell viability of HEI-OC1 influenced by neomycin and CDR. (**F**) Six essential genes related to hair cell function were compared using real-time qPCR between the pre- and post-treatment groups.

An increasing body of research has suggested that the excessive production and accumulation of intracellular reactive oxygen species (ROS) in the cochlea is strongly associated with acquired SNHL, especially aminoglycoside-induced ototoxicity [41,42]. In addition, ROS could activate programmed cell death pathways, such as apoptosis and ferroptosis, thereby reducing the viability of cochlear cells [43,44]. In our results, pertaining the ROS levels, the addition of neomycin, an aminoglycoside ototoxic antibiotic, caused a significant increase in the ROS level, which was confirmed by the flow cytometry and fluorescent microscopy results (Figure 8A,B). However, the addition of CDR, the medicinal cocktail released by our conductive hydrogel, rescued the oxidate stress. Similarly, the aberrantly elevated percentage of apoptotic HEI-OC1 cells induced by neomycin was recovered by CDR drugs (Figure 8C,D). On the other hand, the results of the cell viability demonstrated a similar trend (Figure 8E). The chosen dosage of neomycin was determined through a gradient concentration of neomycin (Figure S5A,B).

### 3.8. Drugs Released by Conductive Hydrogel Upregulates Hair Cell Function-Related Genes

In order to assess whether the drug cocktail released by conductive hydrogel could rescue hair cell function from a transcriptomic level, qPCR was conducted on several closely related genes. As shown in Figure 8F, all the chosen genes exhibited a similar regulation pattern, in which neomycin downregulated them but CDR upregulated them. Among these six genes, i.e., CALB1 (inner hair cell-related), Prestin (outer hair cell-related), Atoh1 (proneural transcription factor), BDNF (neurotrophic factor), and CALM1 (transduction-related), Prestin and BDNF genes exhibited a greater extent of upregulation when compared to the rest. 

### 3.9. Drug Cocktail Released by Conductive Hydrogel Protects Hair Cells from Ferroptosis

**Figure 9:**
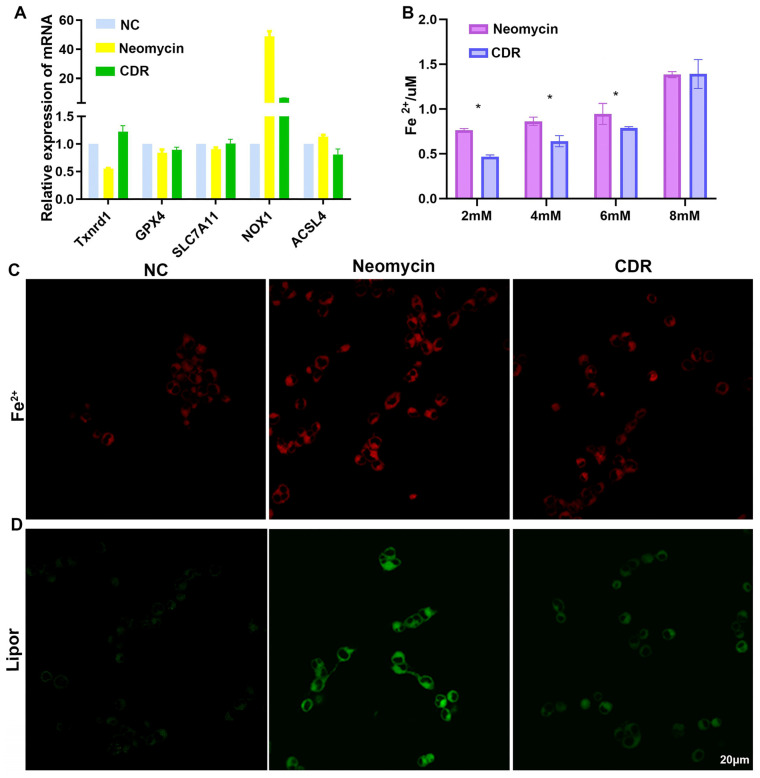
Protection of HEI-OC1 hair cells from ferroptosis. (**A**) Essential ferroptosis-related genes affected by CDR treatment using real-time qPCR. (**B**,**C**) Fe^2+^ accumulation quantified using colorimetric assay and fluorescent probe. (**D**) Lipid peroxidation assessed using fluorescent probe. * *p* < 0.01.

As mentioned in the above section, ferroptosis is a form of regulated cell death linking redox biology and auditory diseases [44]. The initiation and execution of ferroptosis lies at the intersection of amino acid and glutathione metabolism, lipid metabolism, iron metabolism, and other pathways. After the optimal concentration of neomycin was determined using a gradient analysis (Figure S5C–E), the changes in the two essential biomarkers for ferroptosis, i.e., Fe^2+^ accumulation and lipid peroxidation, was quantified using a colorimetric assay and fluorescent probe. As shown in Figure 9B–D, neomycin increased the level of Fe^2+^ and lipid peroxides, whereas the CDR cocktail rescued this ferroptosis-related damage. In addition, further transcriptomic analysis was performed to elucidate the molecular mechanism underlying the CDR-induced anti-ferroptotic effect of hydrogel-released medications (Figure 9A). The ferroptosis-inhibiting genes, e.g., GPX4 (chief regulator of ferroptosis) and TXNRD1 (thiol redox-related antioxidant) were upregulated by the CDR treatment, whereas the ferroptosis-promoting genes, e.g., ACSL4 (long-chain free fatty acid convertor), SLC7A11 (cysteine importer), and NOX1 (local generator of ROS) were downregulated. These results highlighted the role of ferroptosis as a nexus connecting redox biology, metabolism, and otological disease.

### 3.10. Potential Molecular Mechanism Underlying the Hydrogel-Facilitated, Medicinal Cocktail-Induced Otoprotective Effects

**Figure 10:**
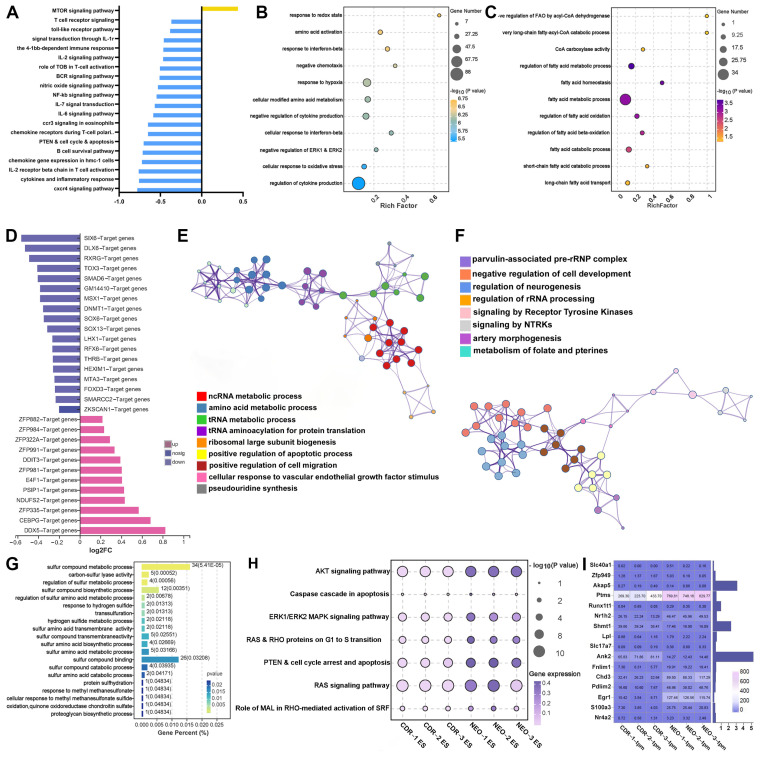
Bioinformatic analysis of differentially expressed genes pre- and post-CDR treatment of hair cells. (**A**) GSVA analysis of DEGs, with color coding clearly demarcating upregulation and downregulation of genes. (**B**) Bubble plot of GO analysis in general. Each bubble represents an enriched pathway, the size of which indicates the gene count within the pathway, and the color intensity reflects the significance level of enrichment. (**C**) Bubble plot of GO analysis based on fatty acid metabolism-related genes. (**D**) Transcription factor-targeted genes identified by JASPER database. (**E**) Cluster GO analysis of transcriptional regulatory network which was concluded by DEGs regulated by ferroptosis-related TF. (**F**) Cluster GO analysis of metabolism pathways based on DEGs regulated by ferroptosis-related TF. (**G**) Histogram of sulfur metabolism pathways, with color intensity of bars indicating significance levels and length representing gene percentage. (**H**) Bubble plot of cell cycle- and apoptosis-related pathways across various samples. Bubble size represents the level of enrichment significance, and the color intensity indicates the pathway activity level in each sample. (**I**) Heatmap illustrating gene expression profiles of metal transport and metabolism across different samples, with each cell’s numerical value reflecting the level of expression in that sample.

Firstly, the violin plots indicated that the distribution of transcripts per million (TPM) among the three groups were comparable (Figure S7A). Our sequencing results identified 2287 differentially expressed genes (DEGs) before and after the CDR cocktail treatment (Figure S7B). A functional enrichment analysis (gene set variation analysis, GSVA) was conducted to reveal the protective mechanism of CDR treatment for aminoglycoside-exposed HEI-OC1 cells (Figure 10). On the one hand, the heatmaps diagrammatized the potential pathways and regulatory factors concluded by the GSVA analysis based on curated and regulatory gene sets (Figure S7C,D). Multiple chemokine and inflammatory pathways such as the interleukin-related ones were found to be significantly downregulated in the CDR group (Figure 10A,B). On the other hand, the bubble plots presented multiple fatty acid metabolism pathways that were regulated by the CDR treatment (e.g., negative regulation of FAO by acyl-CoA dehydrogenase, CoA carboxylase activity, very long-chain fatty-acyl-CoA catabolic process, and fatty acid catabolic process) (Figure 10C). Since fatty acids serve as a direct precursor of many inflammatory mediators, including prostaglandin E2 (PGE2), prostacyclin (PGI2), thromboxane A2 (TXA2), and leukotrienes C4 (LTC4), the inhibition of lipid metabolism by the CDR therapy might have a significant impact on the suppression of the cochlear implantation-induced inflammatory response.

Secondly, to analyze the potential mechanism of the CDR-induced attenuation on the ferroptosis of the hair cells, a trans-acting factors analysis, which was based on the DEGs before and after the CDR treatment, was conducted. The top 30 transcription factors (TFs) predicted to regulate these DEGs were exhibited in a bar chart, and the potential TF set was categorized by domain (Figure 10D and Figure S7E). After screening out the ferroptosis-related TFs (Figure S7F), a GO cluster analysis was performed on the DEGs, which were regulated by these TFs (Figure 10E,F). It revealed that many transcriptional mechanisms, such as ncRNA metabolism, ribosomal large subunit biogenesis, and tRNA aminoacylation process, might be associated with the inhibition of ferroptosis (Figure S8). In addition, a receptor tyrosine kinase signaling pathway found by the GO clustering has been reported to be associated with the decreased degradation of GPX4 and consequently a reduced susceptibility to ferroptosis [45,46]. 

Lastly, as two of the three mainstays of ferroptosis, sulfhydryl metabolism- and metal ion-related gene expression were assessed using GO analysis, respectively (Figure 10G,I). Several metal transport-related genes in the CDR group showed a decreased trend. Similarly, the inhibition of lipid metabolism also contributed to the reduction in lipid peroxidation, thereby facilitating the dampened ferroptosis after CDR treatment (Figure 10C and Figure S8). Furthermore, the enrichment analysis also demonstrated the reduced activation of multiple signaling pathways relating to the cell cycle transition and apoptosis (Figure 10H), which might explain the anti-apoptotic effect of the CDR cocktail treatment (Section 3.7).

## 4. Discussion

### 4.1. GelMA/PEDOT:PSS Conductive Hydrogel as a Potentially Suitable Coating for CI Electrode

The rationale for developing a composite hydrogel in this study was to combine the high processability, hydrophilicity, biocompatibility, and biodegradability of GelMA hydrogel with the electroconductivity of PEDOT:PSS polymers [47], which potentially serves as an ideal coating for the intracochlear electrode used by a cochlear implant [16]. Our results demonstrated that the mechanical, electrochemical, and morphological properties of the hydrogel could be tuned by changing the concentration of PEDOT:PSS. In addition, the beneficial changes in the material properties for target clinical application did not sacrifice the biocompatibility and biosafety of the composite conductive hydrogel. 

Firstly, considering its electrobiological properties, our GelMA/PEDOT:PSS conductive hydrogel would serve as a remarkable coating for a CI electrode as it facilitates electrical stimulation while offering cytocompatibility with cochlear hair cells and neurons. The fundamental principle of the CI electrode is to directly stimulate the SGNs while bypassing the dysfunctional hair cells of the cochlea [16]. Comparing to coatings made of non-hydrogel and non-conductive polymer materials, our composite substrate could act as a promising interface for merging humans and machines [48]. 

Secondly, considering its mechanical properties, the conductive hydrogel-coated electrode could facilitate the fabrication of a ‘soft electrode’, which potentially achieves the atraumatic intracochlear insertion of an electrode array. Even for a patient with no residual hearing, intact cochlear structures are an important prognostic factor in postoperative hearing performance [49,50]. Properties such as stiffness and flexibility are crucial factors for the insertion and final positioning of electrode arrays in the scala tympani [51]. 

Thirdly, the low in vivo inflammatory response and fibrosis formation induced by our conductive hydrogel supports its long-term intracochlear use. Up to one third of patients may lose their residual hearing within a few months after cochlear implantation [52,53]. The latter two vicious changes increase the impedance of the electrodes, prevent efficient electrical stimulation, and compromise neuronal activity, thereby leading to inconsistent performance during long-term implantation [54]. Our hydrogel alone already has the potential to reduce intracochlear inflammation and fibrosis. 

In summary, holding comparable or even superior material properties to conductive hydrogel coatings developed by other groups [55,56,57], our GelMA/PEDOT:PSS composite hydrogel could potentially serve as a coating for a CI electrode. Future work should include additional electrochemical analysis (e.g., capacitance) and testing in artificial perilymph.

### 4.2. GelMA/PEDOT:PSS Conductive Hydrogel as an Adequate Reservoir for Intracochlear Drug Delivery

To the best of our knowledge, we were the first to develop a conductive hydrogel-based local delivery platform for the sustained release of the CDR drug cocktail containing CHIR, RepSox, and DAPT. Our results demonstrated that the compact composite hydrogel could load sufficient cargo potent enough to elicit otoregenerative and otoprotective effects (Section 4.3 and Section 4.4). 

Firstly, our GelMA/PEDOT:PSS conductive hydrogel could facilitate the CI electrode-based intracochlear delivery of medications. Prior to the CI era, drug delivery to the inner ear was facing considerable challenges in the treatment of inner ear diseases, and was mostly limited to systemic and intratympanic administrations [58]. On the other hand, since the discovery and in vitro usage of the aforementioned otological medications several years ago [13,59,60,61], one of the main reasons preventing them from being used in the mammalian cochlea has been the difficulty in in vivo delivery. 

Secondly, the loading and release of our medicinal cocktail could be closely linked to various material properties of the conductive hydrogel (Section 4.1) [36]. The conductive hydrogel could be loaded with a macromolecule or small molecule drugs through chemical and physical drug–polymer interactions ranging from covalent conjugation to secondary interactions such as electrostatic and hydrophobic associations [62]. On the other hand, conductive hydrogels could release drugs through network degradation, swelling, and mechanical deformation [63,64].

Thirdly, drug release from the GelMA/PEDOT:PSS conductive hydrogel coating could be improved by the built-in electrical stimulation of the CI electrode. As a prerequisite, chronic electrical stimulation in partial hearing candidates does not adversely affect functioning residual hair cells and SGNs [65]. In addition, conductive hydrogel-coated electrodes offer electrochemical advantages over palatinum electrodes while maintaining neural function, despite negligible coating loss during chronic electrical stimulation [66,67].

In summary, our GelMA/PEDOT:PSS composite hydrogel could serve as an adequate reservoir for CI electrode-based intracochlear drug delivery. Future work should include optimizing the release of an individual drug in the cocktail and developing multi-stimuli-responsive hydrogels.

### 4.3. Conductive Hydrogel-Released Drug Cocktail Possesses Otoregenerative Potential

Several studies have discovered that supporting cells in the cochlea possess the capacity to undergo trans-differentiation into hair cells [59,60,61,68]. This is mainly because among supporting cells, there are some subpopulations of cells with stem/progenitor cell-like properties, which are identified by markers such as Lgr5, Sox2, and Cdkn1b [69,70]. The small-molecule drug cocktail used to achieve this purpose adopts the principle for induced pluripotent stem cell (iPSC) reprogramming through the manipulation of various signaling pathways, such as Wnt, Notch, and TGF-β cascades [71,72,73,74]. Using a murine cochlear organoid [75], drugs released from our GelMA/PEDOT:PSS conductive hydrogel could not only support the proliferation of cochlear stem cells but also push the supporting cells to differentiate into hair cells through regulating dozens of cochlear microenvironment-associated genes.

Firstly, conductive hydrogel-released drugs could provide a comparable otoregenerative effect to as-received drugs, but with possibly material-induced differences, especially in the maintenance of cell stemness. Compared to the positive control, the conductive hydrogel group resulted in a lower amount of Lgr5^+^ supporting cells but upregulated Sox2 expression (Section 3.5). The mechanism underlying this differential effect could be multifactorial. On the one hand, many features of biomaterials have been established to be critical factors in influencing the phenotype of stem cells [76,77]. On the other hand, the trans-differentiation of supporting cells into hair cells might occur through mechanisms other than Lgr5 positivity [78]. Sox2 is also widely regarded as a common marker for progenitor cells in the cochlea, which could be transformed into hair cells [79]. Furthermore, the recipient ratio of every drug in the cocktail might be different between the conductive hydrogel group and positive control [80].

Secondly, the conductive hydrogel and its released drugs could alter the transcriptomic profile of the cochlea by differentially regulating multiples genes involved in various aspects of cochlear homeostasis and auditory function. On the one hand, few transduction-related genes (e.g., CDH23, CLAM1, and TMC1) were upregulated. CDH23 acts as the tip link of a single stereocilium [81], whereas CALM1 forms a cap on the cytoplasmic surface of the TMC1 ion channel complex [82] in the hair cell molecular mechanotransduction machinery. On the other hand, several synaptic and neurotransmitter genes (e.g., Ribeye, VGlut3, and Chrna9) were also upregulated. VGlut3 is strongly expressed in inner hair cells, and associated with noise-induced ototoxicity and the repair of cochlear ribbon synapses [83,84]. Ribeye is the main protein involved in the assembly of presynaptic ribbon synapse constituents, vesicle recruitment, and synapse maturation [85]. The cochlear ribbon synapse defines the synaptic junction established between the terminations of the auditory nerve and the inner hair cells, thereby serving as a crucial player in the encoding of auditory data [86]. 

Thirdly, the GelMA/PEDOT:PSS conductive hydrogel might serve as a substitute or even a superior replacement for Matrigel used in the cochlear organoid. Organoids mimic real organs by recapitulating many biological features including the spatiotemporal organization of tissue-specific cells, cell–cell interactions, cell–matrix communications, and certain physiological functions, making them promising models for the research of organ development, function, and disease treatment [75]. Although Matrigel, a hydrogel made of basement membrane from natural ECM, has been used for over a decade in 3D organoid systems, its several disadvantages have prevented it from being used in vivo, especially in a human otological context [87,88]. Therefore, one can either modify and enhance Matrigel by incorporating other biomaterial [89], or completely replace Matrigel with another 3D biomaterial [90], just like our composite conductive hydrogel with decent mechanical and electrochemical properties and satisfactory biocompatibility and biosafety.

In summary, our hydrogel-based drug delivery platform strongly supports the trans-differentiation of supporting cells into hair cells. Future work should include optimizing the conductive hydrogel into a reliable matrix for a cochlear organoid and developing drug cocktails regulating pathways other than the Wnt and Notch cascades for improved otoregenerative potential. 

### 4.4. Conductive Hydrogel-Released Drug Cocktail Exerts Otoprotective Effect

Auditory hair cell death is a common cellular cause for acquired SNHL with various etiologies, such as ototoxic drugs, noise exposure, and aging. Programmed cell death (PCD) plays a critical role in the development and diseases of the inner ear. The conventional pathways of PCD (e.g., apoptosis and autophagy) and nearly a dozen new non-apoptotic cell death subroutines (e.g., ferroptosis and pyroptosis) have all become targets for otoprotective strategies [91]. For the first time, we demonstrated that a CDR drug cocktail released by the conductive hydrogel could exert a dual protective effect on hair cells from apoptosis and ferroptosis. 

Firstly, the GelMA/PEDOT:PSS conductive hydrogel-based drug delivery could exert an anti-oxidative and anti-apoptotic effect while upregulating hair cell function-related genes possibly through previously under-investigated signaling pathways. The excessive generation of ROS in the cochlea is often triggered by exposure to loud sound and ototoxic medications, and can then lead to caspase-mediated PCD by apoptosis [92,93]. Targeting the oxidative stress and apoptosis, antioxidants (e.g., N-acetyl cysteine, Ebselen, and salicylate) and antiapoptotic agents (e.g., z-VAD-fmk and z-LEHD-fmk) have been developed as otoprotective pharmacotherapies, respectively [91]. However, the CDR drug cocktail (i.e., CHIR, DAPT, and Repsox) used in our study works through different mechanisms, according to previous studies [13]. For example, CHIR functions as a Wnt pathway activator, DAPT as a Notch pathway inhibitor, and Repsox as a TGF-β pathway inhibitor [94,95,96]. Therefore, it is not surprising that our CDR drug cocktail can protect hair cells from apoptosis. 

Secondly, the GelMA/PEDOT:PSS conductive hydrogel-based drug delivery could exert anti-ferroptotic effect by ameliorating the aminoglycoside-induced accumulation of ferrous ions and lipid peroxides. Ferroptosis is a unique form of non-apoptotic regulated cell death that is dependent on iron and ROS, and is characterized by excessive lipid peroxidation downstream of metabolic dysfunctions [97,98]. However, ferroptosis-oriented studies relevant to inner ear disease are very scarce, most of which have focused on cisplatin-induced ototoxicity [99,100,101]. None of these strategies are directly linked to our CDR cocktail.

Thirdly, the otoprotective effect rendered by hydrogel-based drug delivery might be caused by a network of metabolism-related signaling pathways. On the one hand, cell metabolism acts as an essential link between cell growth and apoptosis. As discovered by our sequencing analyses, metabolic pathways and apoptotic pathways have both been tightly related [102,103]. Conversely, nucleotide-based metabolic intermediates released from apoptotic cells, such as purine, pyrimidine, and pterin, can also act as tissue messengers to suppress inflammation and boost cell proliferation in healthy neighboring cells [104]. On the other hand, the signaling pathways governing metabolism and ferroptosis are inextricably linked [98,105,106]. In addition, the CDR treatment-induced anti-inflammatory effect on hair cells might be related to downregulated pathways signaling chemokines (associated with arachidonic acid metabolism) [107,108] and iNOS (governed by arginine, lysine, and nitrogen metabolism) [109,110].

In summary, our hydrogel-based drug delivery platform exerts a dual protective effect on hair cells from apoptosis and ferroptosis. Future work should explore whether the CDR cocktail has an effect on other forms of PCD, such as autophagy and pyroptosis, and verify whether CDR can activate and regulate other signaling pathways, such as Hedgehog, NF-κB, and Akt pathways.

## 5. Conclusions and Future Perspectives

In this proof-of-concept study, we are the first to systematically demonstrate that the composite GelMA/PEDOT:PSS conductive hydrogel could support the sustained release of a multifunctional drug cocktail containing CHIR, DAPT, and Repsox. Considering its remarkable material properties and biocompatibility, our hydrogel platform could not only serve as a suitable coating for the cochlear alloy electrode, but also elicit a relatively low inflammatory response and fibrosis formation after implantation. Using the cochlear organoids and cochlear cells as experimental models, we then established that the conductive hydrogel-released drug cocktail possesses otoregenerative potential and an otoprotective effect. On the one hand, medications released from the hydrogel could provide a comparable effect to as-received drugs with material-induced differences, alter the transcriptomic profile by differentially regulating multiple genes involved in cochlear homeostasis and auditory function, and function as a potential alternative to Matrigel used in the cochlear organoid. On the other hand, the conductive hydrogel-based drug delivery could exert an anti-oxidative and anti-apoptotic effect while upregulating hair cell function-related genes, provide anti-ferroptotic effect by ameliorating the aminoglycoside-induced accumulation of ferrous ions and lipid peroxides, and potentially influence myriads of signaling pathways including some previously under-investigated signaling cascades. In conclusion, our GelMA/PEDOT:PSS conductive hydrogel-based delivery system could simultaneously restore dysfunctional hair cells responsible for high-frequency hearing loss and protect hair cells responsible for residual low-frequency hearing, thereby boosting the applicability and broadening the indication of auditory systems such as the bimodal electroacoustic stimulation using both a cochlear implant and hearing aid. Based on the material, transcriptomic, protein, cellular, and organoid data from this study, the ideal next step would be preparation for an animal study and human clinical trials. 

## Data Availability

All data generated or analyzed during this study are included in this article.

## Supplementary Materials

The following supporting information can be downloaded at: https://www.mdpi.com/article/10.3390/biom14010095/s1, Figure S1. Fabrication of GelMA and GelMA/PEDOT:PSS hydrogels; Figure S2. Biocompatibility and biodegradation of GelMA and composite conductive hydrogel; Figure S3. Cellular biosafety as manifested by cell survival and death of neuronal cells and cochlear cells; Figure S4. Determination of an optimal concentration of CDR medicinal cocktail released by the GelMA/PEDOT:PSS conductive hydrogel using cochlear organoids; Figure S5. Establishment of an aminoglycoside-induced ototoxicity model; Figure S6. Free of systemic toxicity of various hydrogels when implanted in vivo; Figure S7. Bioinformatic analysis of differentially expressed genes (DEGs) pre- and post-CDR treatment of cochlear hair cells; Figure S8. Bioinformatic analysis of metabolism-related pathways and intermediate metabolites affected by the CDR treatment of cochlear hair cells; Video S1: 10% GelMA hydrogel exhibits relatively better mechanical features during manual handling; Video S2: 7% GelMA hydrogel exhibits relatively worse mechanical features during manual handling.
