# Supplementary material for: GelMA/PEDOT:PSS Composite Conductive Hydrogel-Based Generation and Protection of Cochlear Hair Cells through Multiple Signaling Pathways
Source: Biomolecules. 2024 Jan 11;14(1):95. doi: 10.3390/biom14010095 (PMC10812993; doi:10.3390/biom14010095)

Supplementary Figure legends

Figure S1. Fabrication of GelMA and GelMA/PEDOT:PSS hydrogels. (A) Chemical formula of how gelatin was substituted by methacrylate to generate GelMA and its reaction conditions. (B) The GelMA solution was dialyzed against deionized water using a dialysis bag of 12-14 kDa for 1 week at 40 °C to remove unreacted excess methacrylic anhydride. (C) Freeze-drying of the GelMA solution for 4 days to obtain fibrous white foam. (D) & (E) Macroscopic morphology of pure GelMA and composite hydrogels before and after light curing, respectively. The reaction was initiated by irradiating hydrogel with 365 nm UV light for a total duration of 90 mins. The transparency of hydrogel is getting darker as the content of conductive polymer increases.

Figure S2. Biocompatibility and biodegradation of GelMA and composite conductive hydrogel. (A) Cochlear cell viability when exposed to various biomaterials. (B) *In vitro* biodegradation of various hydrogels. (C) *In vivo* biodegradation of various hydrogels. (D) H&E staining, Masson staining and Hoechst staining of tissue interaction with hydrogel specimens implanted subcutaneously.

Figure S3. Cellular biosafety as manifested by cell survival and death of neuronal cells and cochlear cells. (A) The live/dead assay of PC12 neuronal cells. (B) The live/dead assay of HEI-OC1 cochlear cells. (C) The cell apoptosis assay of PC12 neuronal cells. (D) The cell apoptosis assay of HEI-OC1 cochlear cells.

Figure S4. Determination of an optimal concentration of CDR medicinal cocktail released by the GelMA/PEDOT:PSS conductive hydrogel using cochlear organoids. PC = positive control. NC = negative control.

Figure S5. Establishment of an aminoglycoside-induced ototoxicity model. (A) The effect of gradient concentrations of neomycin on the accumulation of ROS. (B) The effect of gradient concentrations of neomycin on the cell viability of HEI-OC1 cochlear cells. (C) The effect of gradient concentrations of the level of ferrous ions using colorimetric assay. (D) The effect of gradient concentrations of the ferroptosis-related markers using fluorescent probes. NC = negative control.

Figure S6. Free of systemic toxicity of various hydrogels when implanted *in vivo*. NC = negative control.

Figure S7. Bioinformatic analysis of differentially expressed genes (DEGs) pre- and post-CDR treatment of cochlear hair cells. (A) Violin plot of the distribution of transcripts per million (TPM) among the three groups. (B) Volcano plot of DEGs pre- and post-CDR treatment. (C) Pathways concluded by gene set variation analysis (GSVA) analysis based on curated gene sets. (D) Regulatory factors concluded by regulatory gene sets. (E) Statistics of transcription factor (TF) family which might regulate these DEGs after CDR treatment. (F) Top 10 ferroptosis-related TFs sorted by the number of target genes after CDR treatment.

Figure S8. Bioinformatic analysis of metabolism-related pathways and intermediate metabolites affected by the CDR treatment of cochlear hair cells. (A) All potential metabolism pathways after CDR treatment. (B) All potential secondary metabolites pathways after CDR treatment.

Figure S1. Fabrication of GelMA and GelMA/PEDOT:PSS hydrogels. (A) Chemical formula of how gelatin was substituted by methacrylate to generate GelMA and its reaction conditions. (B) The GelMA solution was dialyzed against deionized water using a dialysis bag of 12-14 kDa for 1 week at 40 °C to remove unreacted excess methacrylic anhydride. (C) Freeze-drying of the GelMA solution for 4 days to obtain fibrous white foam. (D) & (E) Macroscopic morphology of pure GelMA and composite hydrogels before and after light curing, respectively. The reaction was initiated by irradiating hydrogel with 365 nm UV light for a total duration of 90 mins. The transparency of hydrogel is getting darker as the content of conductive polymer increases.

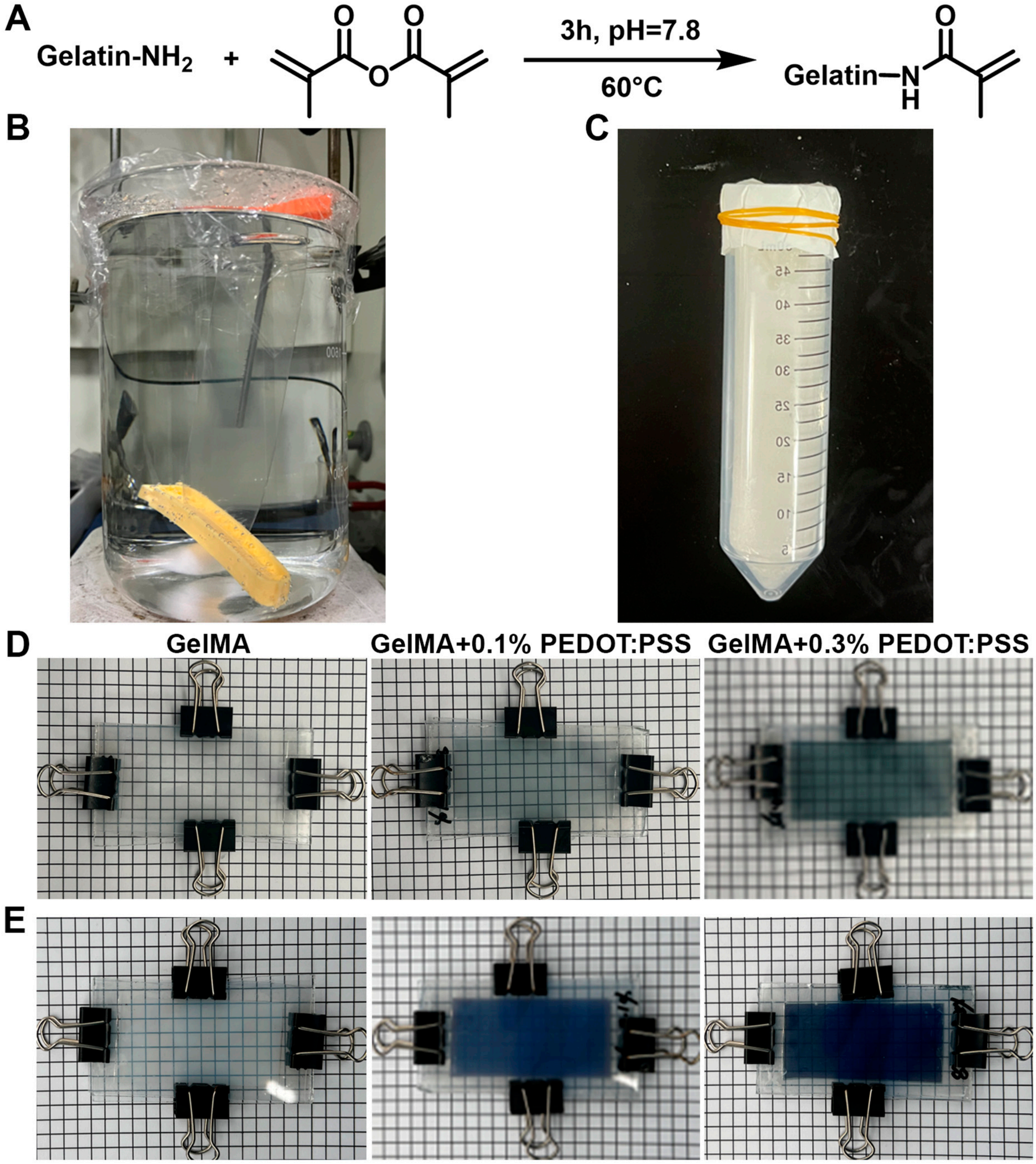

Figure S2. Biocompatibility and biodegradation of GelMA and composite conductive hydrogel. (A) Cochlear cell viability when exposed to various biomaterials. (B) *In vitro* biodegradation of various hydrogels. (C) *In vivo* biodegradation of various hydrogels. (D) H&E staining, Masson staining and Hoechst staining of tissue interaction with hydrogel specimens implanted subcutaneously.

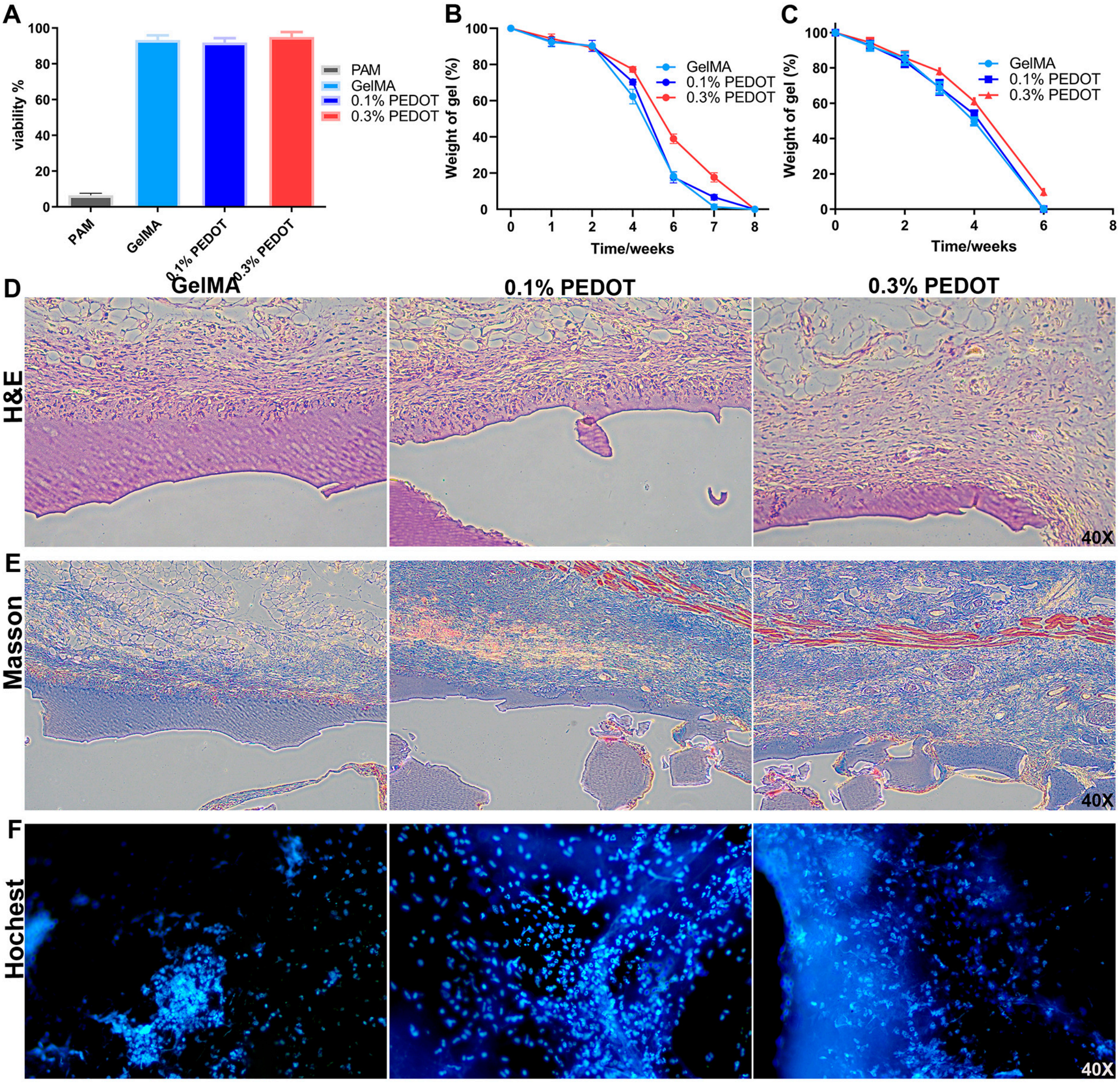

Figure S3. Cellular biosafety as manifested by cell survival and death of neuronal cells and cochlear cells. (A) The live/dead assay of PC12 neuronal cells. (B) The live/dead assay of HEI-OC1 cochlear cells. (C) The cell apoptosis assay of PC12 neuronal cells. (D) The cell apoptosis assay of HEI-OC1 cochlear cells.

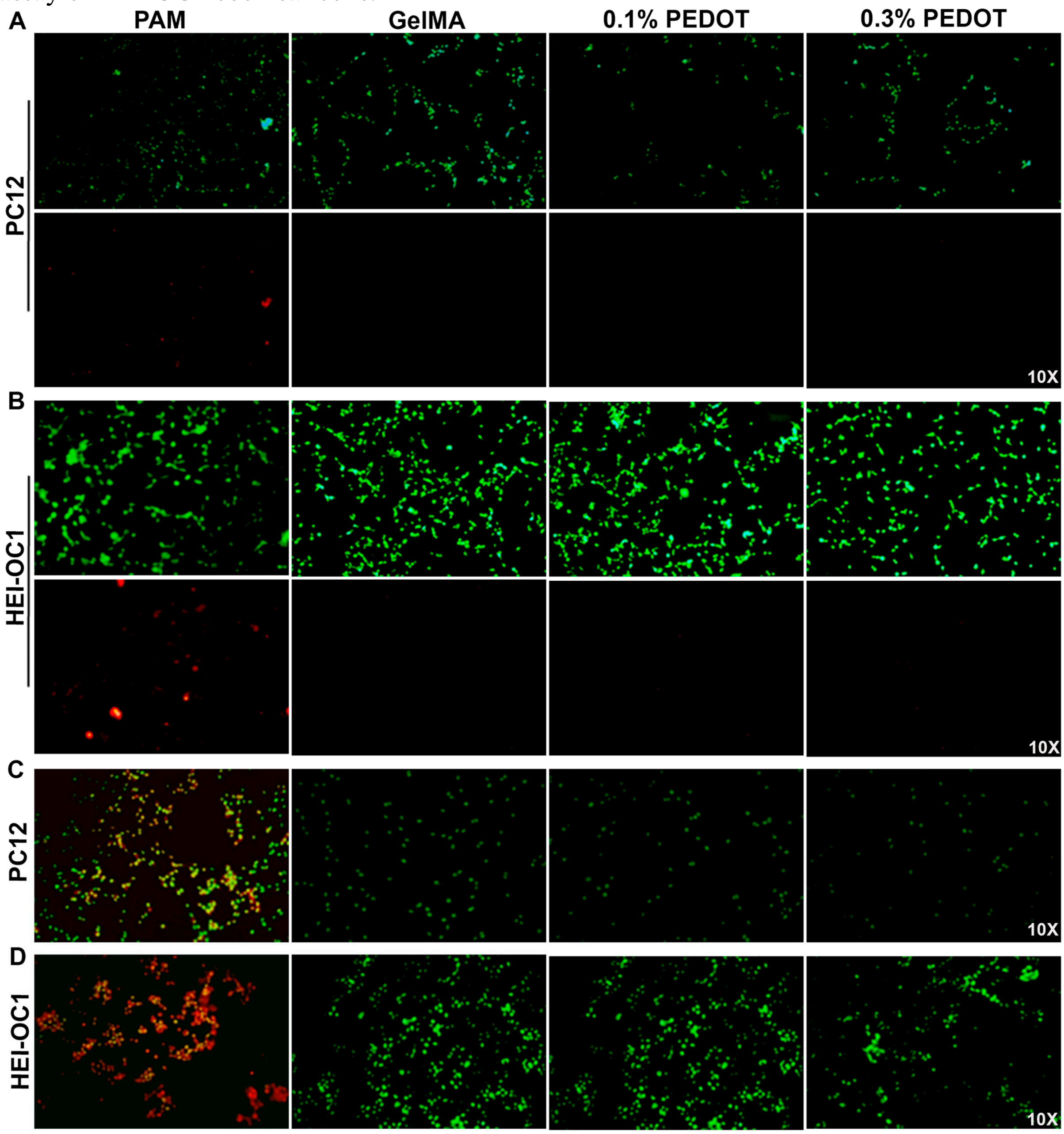

Figure S4. Determination of an optimal concentration of CDR medicinal cocktail.

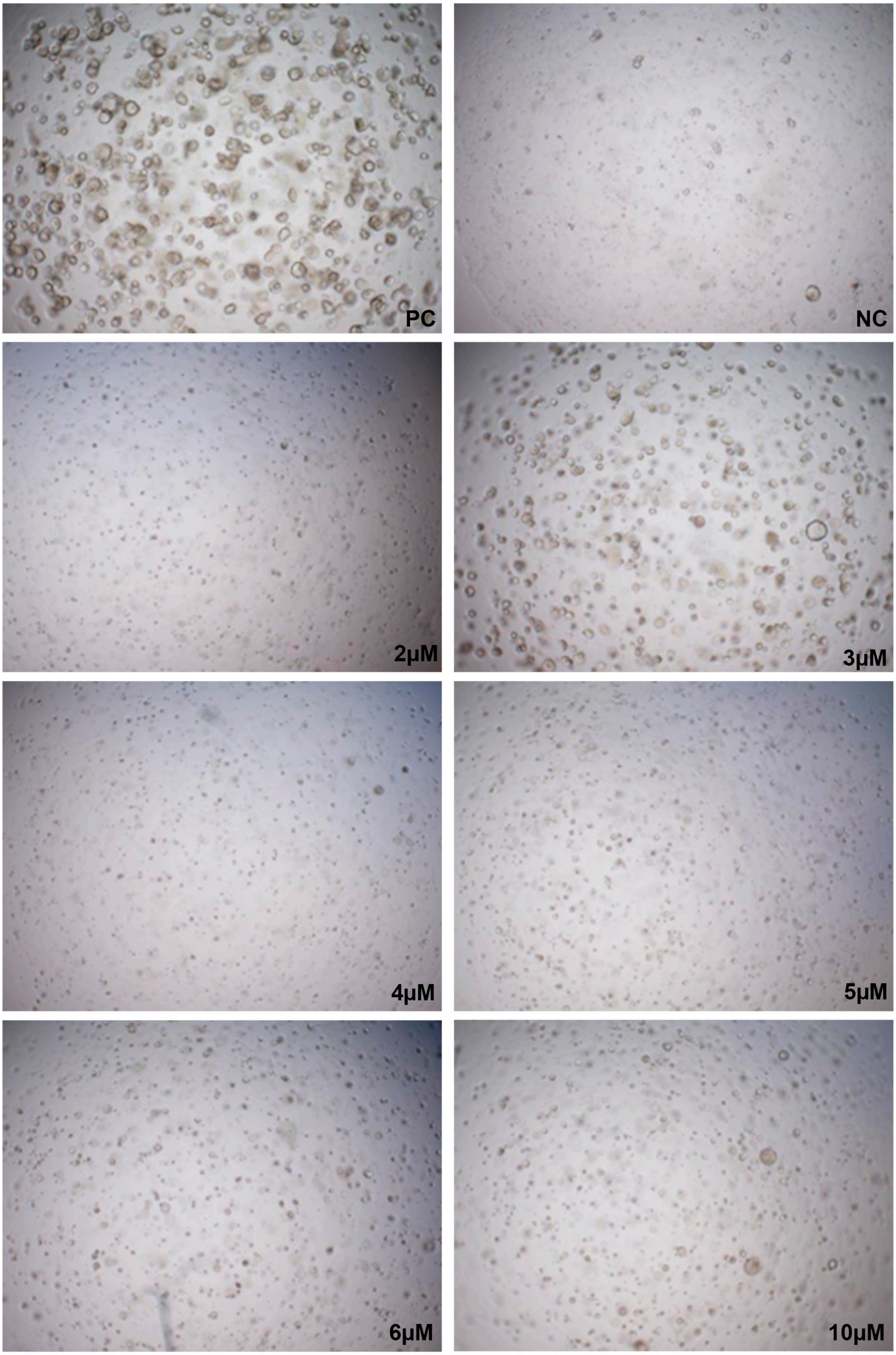

Figure S5. Establishment of an aminoglycoside-induced ototoxicity model. (A) The effect of gradient concentrations of neomycin on the accumulation of ROS. (B) The effect of gradient concentrations of neomycin on the cell viability of HEI-OC1 cochlear cells. (C) The effect of gradient concentrations of the level of ferrous ions using colorimetric assay. (D) The effect of gradient concentrations of the ferroptosis-related markers using fluorescent probes. NC = negative control.

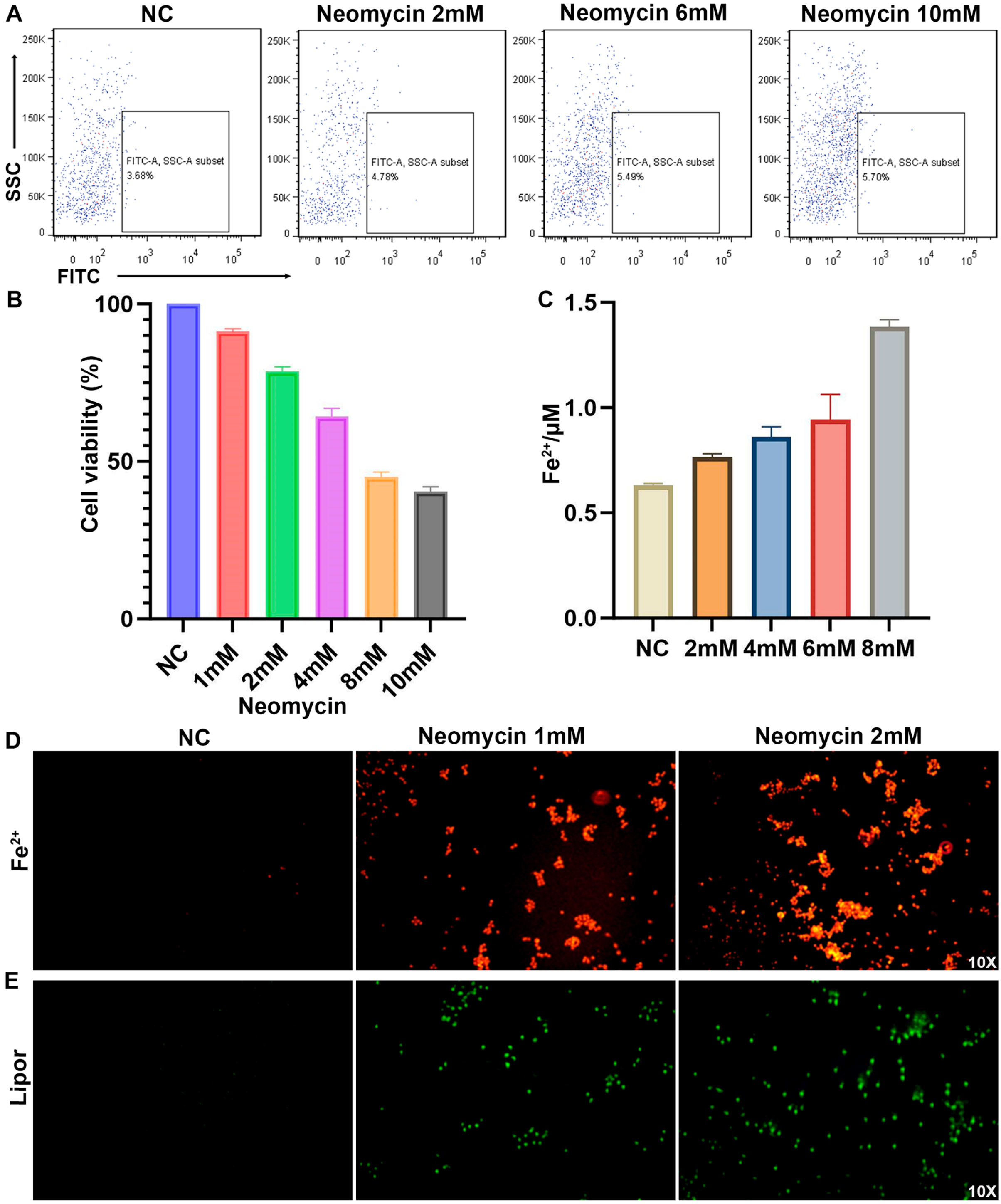

Figure S6. Free of systemic toxicity of various hydrogels when implanted *in vivo*. NC = negative control.

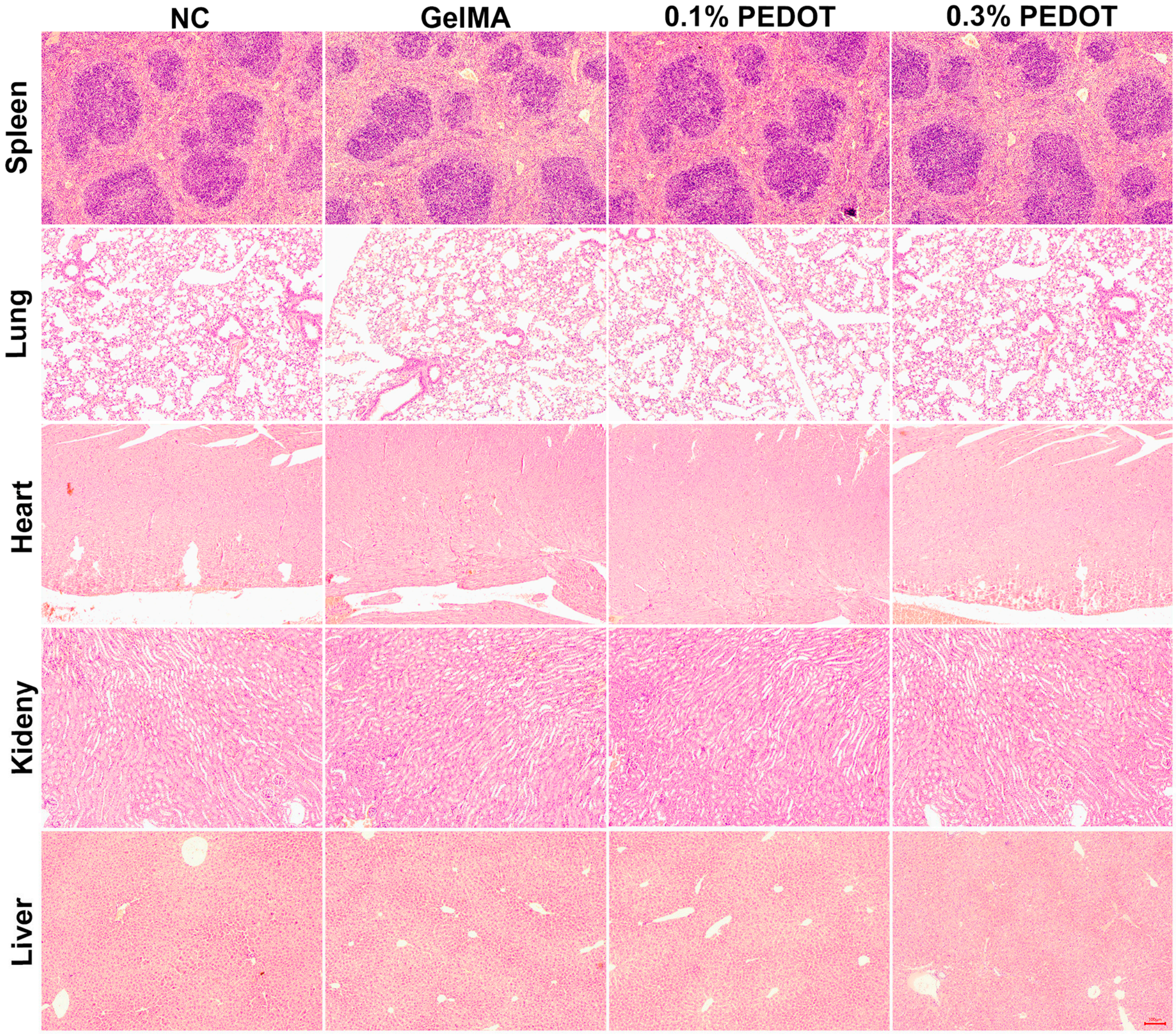

Figure S7. Bioinformatic analysis of differentially expressed genes (DEGs) pre- and post-CDR treatment of cochlear hair cells. (A) Violin plot of the distribution of transcripts per million (TPM) among the three groups. (B) Volcano plot of DEGs pre- and post-CDR treatment. (C) Pathways concluded by gene set variation analysis (GSVA) analysis based on curated gene sets. (D) Regulatory factors concluded by regulatory gene sets. (E) Statistics of transcription factor (TF) family which might regulate these DEGs after CDR treatment. (F) Top 10 ferroptosis-related TFs sorted by the number of target genes after CDR treatment.

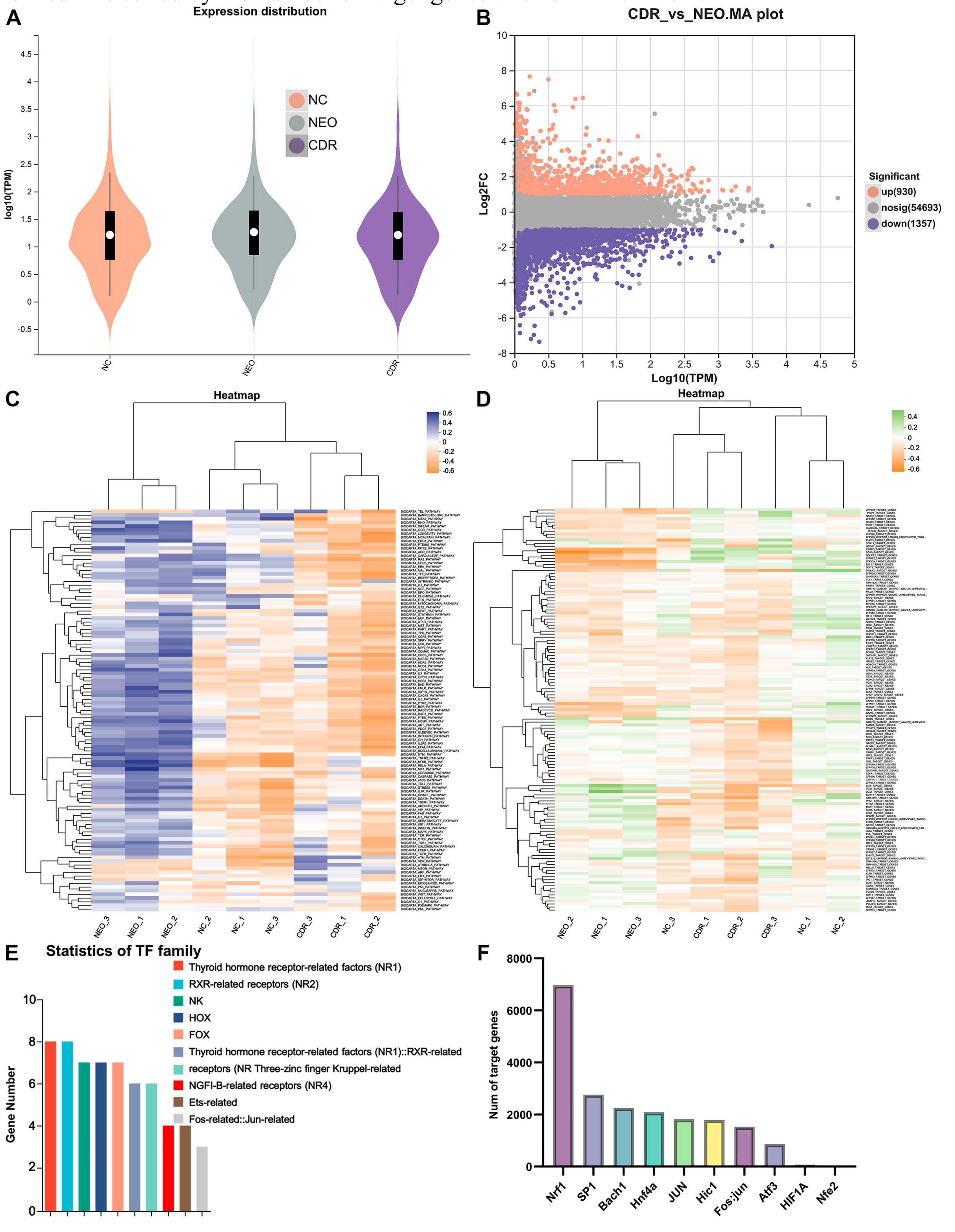

Figure S8. Bioinformatic analysis of metabolism-related pathways and intermediate metabolites affected by the CDR treatment of cochlear hair cells. (A) All potential metabolism pathways after CDR treatment. (B) All potential secondary metabolites pathways after CDR treatment.

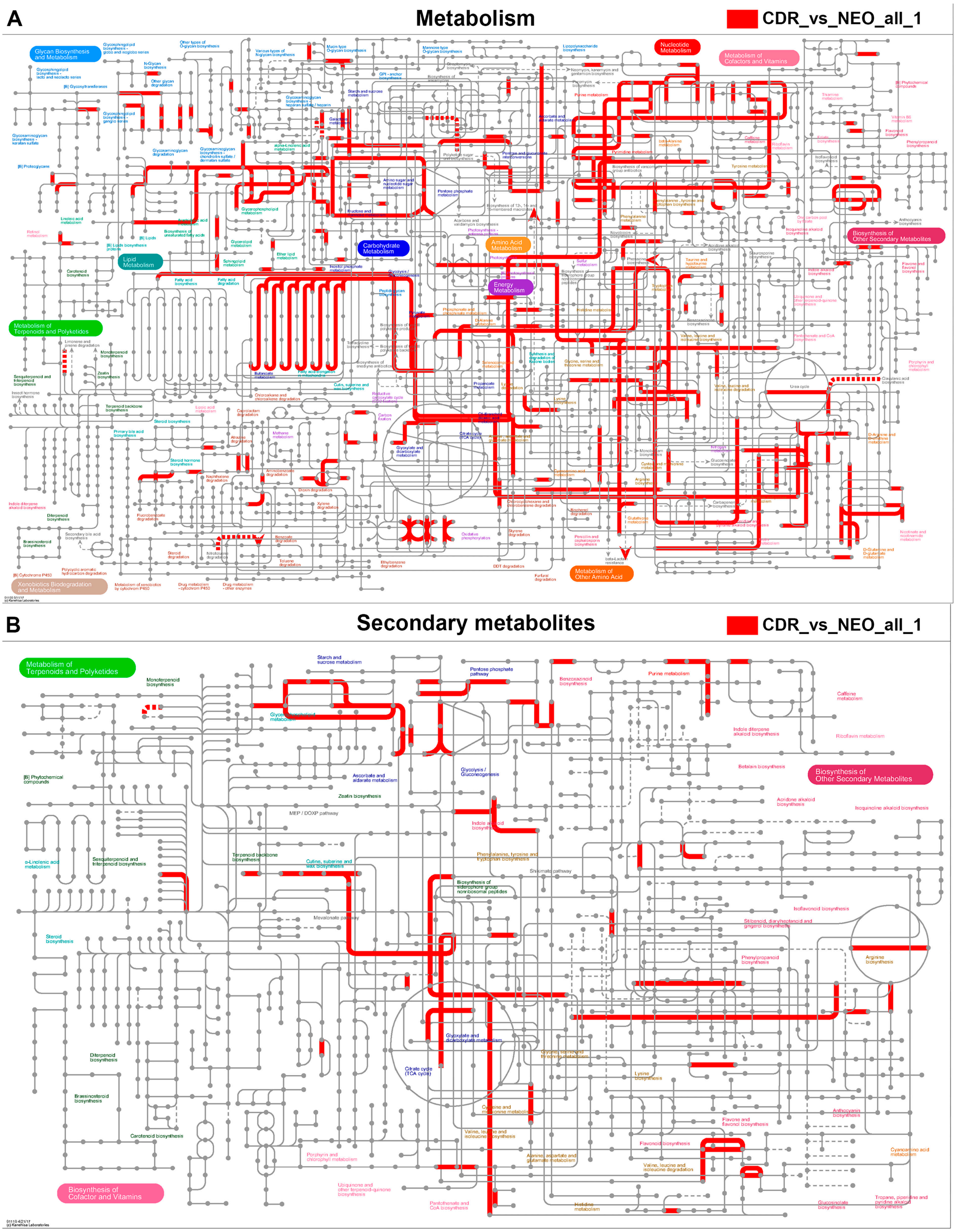

Supplement: Supplementary file 1 [file biomolecules-14-00095-s001.zip › biomolecules-2742203-supplementary.pdf]
